# Evolution of ancient hydrothermal fluids theoretically inverted with initial oxygen isotopes of water

**DOI:** 10.1038/s41598-025-99653-x

**Published:** 2025-05-06

**Authors:** Chun-Sheng Wei, Zi-Fu Zhao

**Affiliations:** State Key Laboratory of Lithospheric and Environmental Coevolution, School of Earth and Space Sciences, University of Science and Technology of China, Hefei, 230026 China

**Keywords:** Geochemistry, Mineralogy, Petrology

## Abstract

While great successes have been achieved during past decades, oxygen isotopic endmembers of water were implicitly adopted by previous studies for quantifying fossil hydrothermal systems worldwide. Both endmember and evolved oxygen isotopes of meteoric (−11.01±0.43 vs. −3.82±0.01‰, one standard deviation of 1SD) and magmatic water (6.57±0.05 vs. 4.21±0.04‰) are, however, theoretically inverted from hydrothermally altered minerals herein. These waters could evolve through binary mixing under isothermal conditions or interacting with susceptible minerals prior to hydrothermal reequilibration. The studied fossil hydrothermal systems can energetically be alive from less than 12 thousand years (Kyr) to 1 million years (Myr). In the viewpoint of thermodynamics and kinetics, caution should be paid to straightforward quantifying hydrothermal systems with the endmember oxygen isotopes of water. Nevertheless, the complexity and diversity of ancient hydrothermal fluids scientifically and methodologically exemplified at the oxygen isotopic level would be secular scenarios during the geodynamic evolution of the continental lithosphere.

**Supplementary Information:**

The online version contains supplementary material available at 10.1038/s41598-025-99653-x.

## Introduction

The liquid water at the Earth’s surface was evidenced by zircons at least from 4.3 to 4.4 Gyr ago^1,2^. Owing to the scientific and practical importance, modern geothermal and/or ancient hydrothermal systems have been drawn great attentions^3–7^. Besides fluid inclusions^8^, ^2^H/^1^H and ^18^O/^16^O (as well as ^17^O/^16^O recently) ratios of hydrothermally altered rocks and/or minerals have been intrinsically utilised for tracking the sources of mobile water and quantifying the respective hydrothermal processes^9–14^. Because of the high analytical precision and reliable fractionation between constituent minerals and water, oxygen isotopes of hydrothermally altered minerals are thus much more ideal and robust to quantitatively unravel the hydrothermal systems.

The physicochemical boundary conditions inside the ‘black-box’ of hydrothermal systems like temperature, initial oxygen isotopes of water and rock, water/rock (W/R) ratio, nature of hydrothermal system (closed vs. open; externally infiltrated vs. internally derived fluid), mechanism of oxygen exchange (diffusion vs. surface-reaction), thermal history (slow vs. rapid cooling) and lifetime (short- vs. long-lived), however, were empirically assumed or qualitatively inferred by most if not all previous studies through the traditional straightforward modelling^15–19^. These uncertainties seriously limit the insightful quantification of active geothermal and/or fossil hydrothermal systems with thermodynamic and/or kinetic confidence from the final observations.

**Fig. 1:**
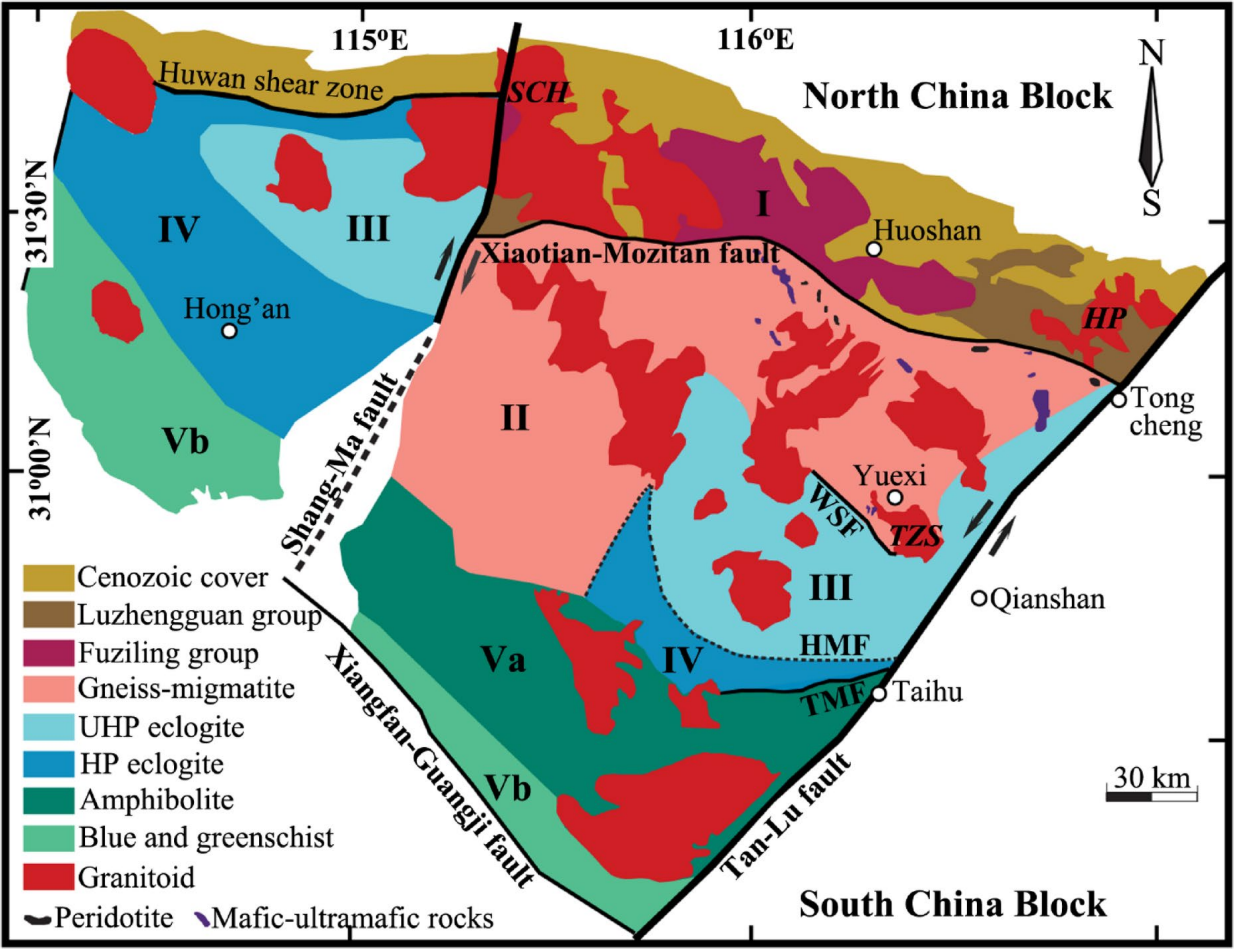
Geological sketch of the Dabie orogen in central-eastern China modified from Refs^25–27^. In terms of field relation and lithotectonic assemblage, geologic units bounded with faults were divided. Traditionally, the western portion beyond the Shang-Ma fault was termed Hong’an (or Xinxian) Block. The Dabie Block (DBB) is bounded by the Shang-Ma fault in the west and the Tan-Lu fault in the east. From north to south, the DBB was further subdivided into five belts: (I) the Northern Huaiyang volcanic-sedimentary belt (flysch series); (II) the Northern Dabie gneissic and migmatitic belt; (III) the Central Dabie ultrahigh pressure (UHP) metamorphic belt; (IV) the Southern-central Dabie high pressure metamorphic belt; and (V) the Southern Dabie intermediate- to low-grade metamorphic belt, respectively. WSF=Wuhe-Shuihou fault, HMF=Hualiangting-Mituo fault, and TMF=Taihu-Mamiao fault. Boldface italic capital letters denote the abbreviations of studied pluton and batholith, for other details refer to Table S1. This figure was generated with Adobe Photoshop CS3 Extended version 10.0 (https://www.adobe.com/cn/products/photoshop.html ).

**Fig. 2:**
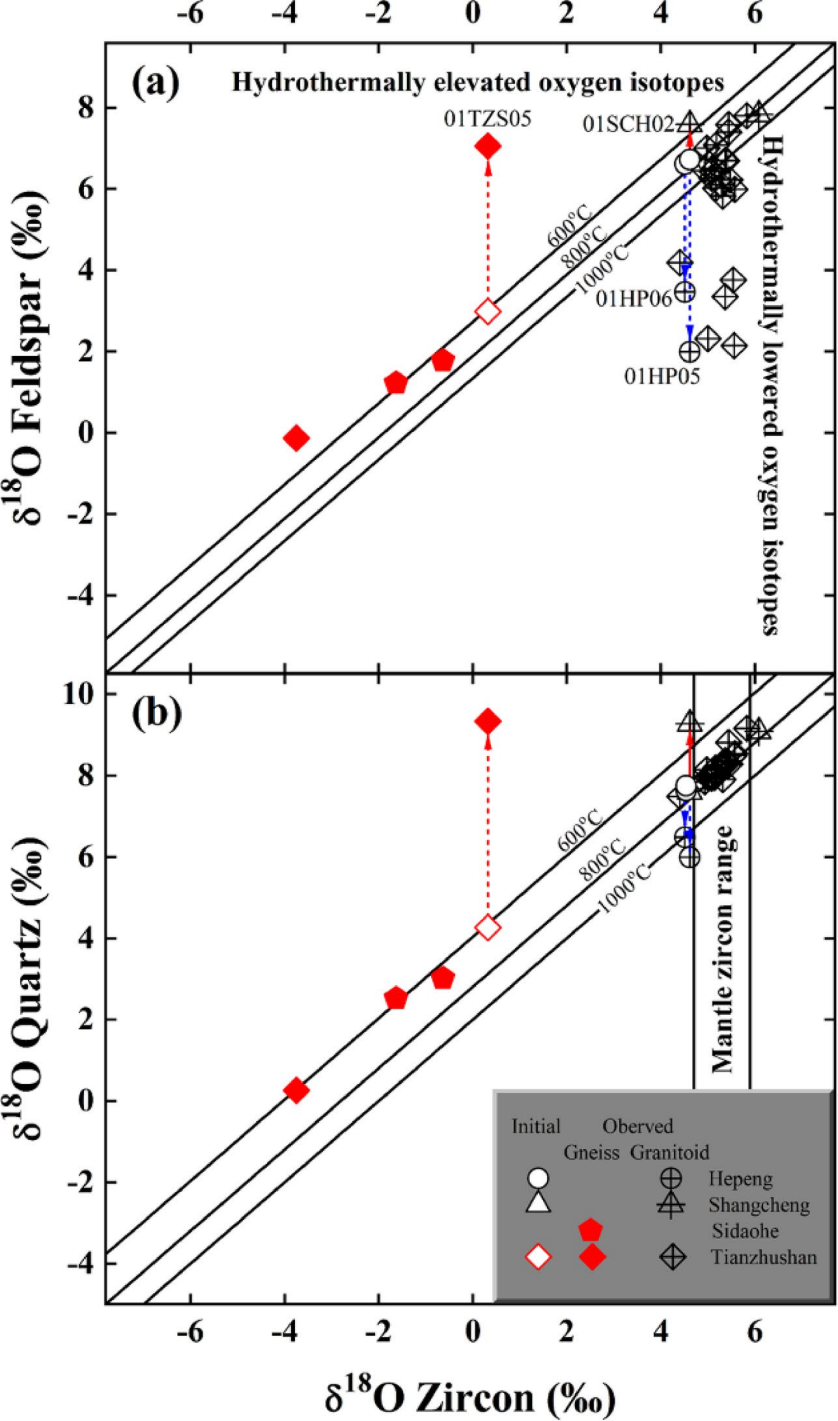
Diagrams of zircon vs. alkali feldspar (**a**) and quartz δ^18^O values (**b**) for the granitoid and gneiss across the Dabie orogen. Lines labelled with temperature are isotherms after Ref^28^, and two vertical solid lines in (**b**) denote the mantle zircon δ^18^O ranges for comparison (cf., Ref^29^). Arrowed lines denote samples theoretically inverted in this study, and their observed and initial oxygen isotopes of constituent minerals refer to Table S1 and Table 1, respectively. The error bar is smaller than the symbol size and thus omitted for clarity herein.

**Fig. 3:**
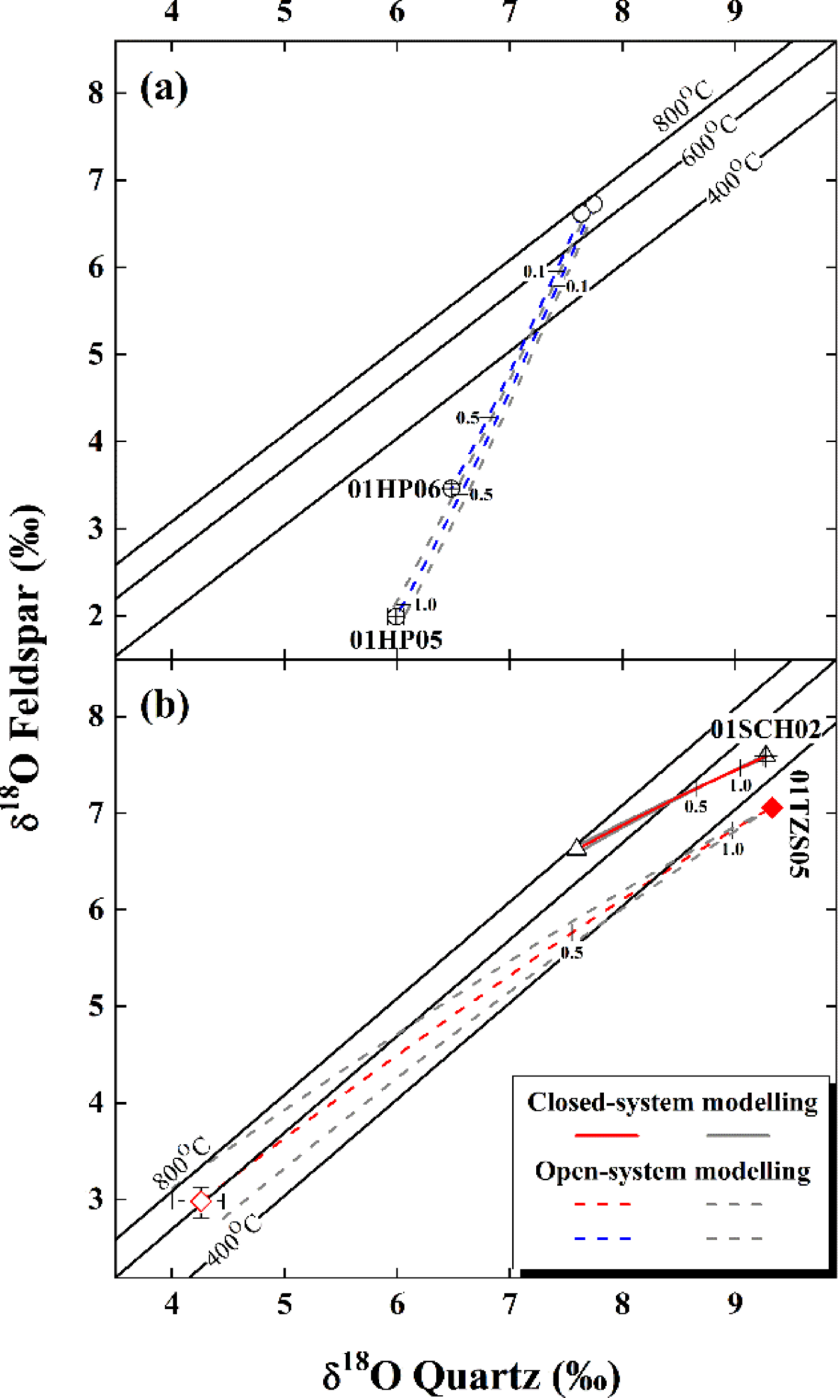
Diagrams of quartz vs. alkali feldspar with concurrently lowered (**a**) and elevated oxygen isotopes (**b**) across the Dabie orogen. Curves with envelopes denote the covaried oxygen isotopes for rock-forming minerals after they were thermodynamically reequilibrated with the ancient meteoric or magmatic water, respectively, whereas small ticks with numbers are W/R ratios. Note that the error bars of the initial oxygen isotopes for rock-forming minerals illustrate their extreme variability rather than 1SD listed in Table 1. For other details refer to Figs. S1 through S4 and Fig. 2.

By contrast, those key parameters outlined above can be theoretically inverted with oxygen isotopes of constituent minerals hydrothermally reequilibrated with water (for other details refer to “Methods” section and Refs^20–24^. For example, $\delta{^\text{18}\text{O}}_{\text{W}}^{\text{i}}$ value (i.e., the initial oxygen isotopes of water prior to the hydrothermal alteration) and W/R ratio will be uniquely determined at a hydrothermal reequilibration temperature from either the closed- or open-system, respectively, below. Since most hydrothermal processes were energetically triggered and/or driven by natural heat engines worldwide, magmatic and metamorphic rocks would be intrinsically desirable for characterising the hydrothermal systems. As a well documented Mesozoic continental orogenic belt, the early Cretaceous postcollisional granitoid and Triassic gneissic country rock are ubiquitously developed across the Dabie orogen in central-eastern China (for other details refer to Fig. 1 and “Methods” section). Moreover, concurrently lowered and/or elevated oxygen isotopes are explicitly observed from the hydrothermally altered rock-forming minerals (see Table S1 and labelled data points in Figs. 2 and 3). These collectively offer an ideal opportunity for quantifying the evolution of ancient hydrothermal fluids distinctively originating from the continental lithosphere.

**Table 1:** Parameters of theoretical inversion for $\delta{^\text{18}\text{O}}_{\text{W}}^{\text{i}}$ values of the externally (E) infiltrated and internally (I) derived water. *Initial quartz ($\delta{^\text{18}\text{O}}_{\text{Qtz}}^{\text{i}}$) and alkali feldspar ($\delta{^\text{18}\text{O}}_{\text{Ksp}}^{\text{i}}$) oxygen isotopes are calculated with the observed zircon δ^18^O values (Table S1) at magmatic or metamorphic temperatures, respectively. The initial oxygen isotopes of samples 01HP05 and 01HP06 are calculated at 740±1^o^C, which is retrieved from oxygen isotopes of quartz-zircon pair from sample 01HP04 and a similar magmatic temperature is whereby assumed on the scale of the Hepeng granitoid pluton. For sample 01SCH02, its intitial values are calculated at 765±8^o^C retrieved from sample 01SCH01 and a similar magmatic temperature is assumed on the scale of the Shangcheng granitoid batholith. For sample 01TZS05, its intitial values are calculated at 610±20^o^C, which is bracketed through samples 00DB63, 00DB64 and 01TZS07 and a common thermal regime on orogenic scale is assumed for the gneissic country rock wherein. ^†^Hydrothermal reequilibration temperatures are calculated with the observed oxygen isotopes of quartz-alkali feldspar pairs for studied samples. ^§^Theoretically inverted from the closed system for sample 01SCH02, whereas the open system is adopted for other samples. It is worthwhile pointing out that results of sample 01HP05 are from Refs^22,23^, whereas those of samples 01SCH02 and 01TZS05 are after Ref^24^. ^#^Ratio of exchangeable oxygen content between water and the indicated mineral.

Sample number	$\delta{^\text{18}\text{O}}_{\text{Qtz}}^{\text{i}}$(‰)*	$\delta{^\text{18}\text{O}}_{\text{Ksp}}^{\text{i}}$(‰)*	T (^o^C)^†^	$\delta{^\text{18}\text{O}}_{\text{W}}^{\text{i}}$(‰)^§^
Granitoid				
01HP05	7.74±0.02	6.72±0.02	140±5	−11.01±0.43 (E)
01HP06	7.64±0.01	6.62±0.01	230	−3.82±0.01 (E)
01SCH02	7.60±0.04	6.63±0.03	475±4	6.57±0.05 (I)
**Gneiss**				
01TZS05	4.26±0.14	2.98±0.10	340	4.21±0.04 (E)
$\:{\text{n}}_{\text{Water}}^{\text{O}}/{\text{n}}_{\text{Mineral}}^{\text{O}}$ ^**#**^	1.68	1.93	/	/

## Results

### General remarks of observation

Zircon δ^18^O values of the studied gneisses evidently scatter from − 3.78 to 0.33‰ (for other details refer to “Methods” section, Table S1 and Fig. 2), indicating the least homogenisation of zircon oxygen isotopes for these orthogneisses during the Triassic regional metamorphism across the Dabie orogen. This is broadly consistent with the apparently heterogeneous zircon δ^18^O values observed from metamorphic rocks around world. On the contrary, zircon δ^18^O values of the early Cretaceous postcollisional granitoids cluster around 5.18±0.45‰ (*n*=29) and overlap with the oxygen isotopic ranges of mantle zircon (i.e., 5.3±0.6‰ after Ref^29^). These suggest that the granitoids with uniform zircon δ^18^O values cannot isotopically link to those orthogneisses with heterogeneous zircon δ^18^O values, which are over twofold larger than zircon oxygen isotopic variability of the studied granitoids on the orogenic scale herein (4.11 vs. 1.70‰ in Table S1).

While equilibrium fractionations are well maintained by most of available zircon and quartz oxygen isotopes under magmatic and/or metamorphic conditions, further examination shows that oxygen isotopes of quartz were concurrently lowered with those of alkali feldspar for two studied granitoids from the Hepeng pluton (i.e., samples 01HP05 and 01HP06 labelled in Fig. 2). The concurrent elevation of oxygen isotopes, however, appears for sample 01SCH02 from the Shangcheng granitoid batholith and sample 01TZS05 from the gneissic country rock intruded by the Tianzhushan granitoid pluton (labelled data points in Fig. 2), respectively.

The concurrently lowered oxygen isotopes observed from the studied granitoids could result from the open-system magmatic evolution like the assimilation by a country rock (i.e., AFC process) depleted in ^18^O. The country rocks intruded by the Hepeng pluton are volcanic-sedimentary rocks (Fig. 1), and the sedimentary endmembers are usually enriched rather than depleted in ^18^O. While hydrothermal alterations by the light δ^18^O water like meteoric and/or oceanic water could result in depletion of ^18^O for the volcanic country rocks, their reports are still lacking up to now. Even if volcanic endmembers depleted in ^18^O indeed exist, the extreme homogeneity of zircon δ^18^O values observed on the scale of the Hepeng granitoid pluton (4.51 to 4.64‰ in Table S1) seems inconsistent with the random assimilation of ^18^O-depleted country rocks and then thorough homogenisation of oxygen isotopes of the assimilated magma wherein. Moreover, the less high magmatic temperature of 740±1^o^C quantified for the Hepeng pluton in this study (see footnote in Table 1) also seems less favourable for the assimilation during magma cooling processes because the latent heat is energetically limited with the crystallisation of a cold granitoid (e.g., Ref^30^). Furthermore, the upper continental crusts themselves are suggestively too cold to substantially assimilate oxygen isotopes of the magma for the Hepeng granitoid pluton.

Similar scenario cannot account for the concurrent elevation of oxygen isotopes for sample 01SCH02 from the Shangcheng granitoid batholith. For example, in contrast to sample 01SCH01 with the highest zircon δ^18^O values throughout this study (6.08±0.04‰ in Table S1) and equilibrium fractionations with quartz (Fig. 2b), a less high zircon δ^18^O value of 4.62‰ is observed for sample 01SCH02 with the concurrently elevated oxygen isotopes of rock-forming minerals. These paradoxical characters are fundamentally incompatible with the progressive assimilating process by ^18^O-enriched country rocks on the batholith scale.

Since metamorphic reactions are usually complex and sluggish under solid conditions with and/or without mediated fluid^31^, the more evident elevation of oxygen isotopes of the gneissic country rock studied herein (i.e., sample 01TZS05 labelled in Fig. 2) could alternatively inherit from its protolith with original nonequilibria. Two gneisses from the Sidaohe in the Hong’an Block, however, well attain equilibrium fractionations between zircon and quartz as well as between zircon and alkali feldspar oxygen isotopes (i.e., samples 00DB63 and 00DB64 in Table S1 and Fig. 2). Moreover, the equilibrium fractionation between zircon and quartz oxygen isotopes is also retained for sample 01TZS07 from another gneissic country rock intruded by the Tianzhushan granitoid pluton (Fig. 2b). While their oxygen isotopes among constituent minerals are considerably variable from one locale to another, similar equilibrium fractionations were thermodynamically achieved for samples noted above (especially between zircon and quartz oxygen isotopes in Fig. 2b). Thereby, it is less likely that original nonequilibria of oxygen isotopes were exceptionally inherited from their protoliths both for the resistant quartz and reactive alkali feldspar for sample 01TZS05. In addition, most of available studies showed that the inert Sm-Nd, Lu-Hf and U-Pb radiometric systems were geochronologically reset during the Triassic regional metamorphism across the Dabie orogen^32–38^. Compared to the inert radiometric systems noted above, oxygen is much more mobile. Given that the inert radiometric systems were explicitly reset, there seems no sufficient reason to argue that the active oxygen isotopes of rock-forming minerals could survive the continental deep subduction, isolate themselves from the Triassic regional metamorphism and inherit the original nonequilibria from their protoliths.

Collectively, the concurrently lowered and/or elevated oxygen isotopes of rock-forming minerals observed in this study are best attributed to the hydrothermal alteration during the postmagmatic or exhumation processes of the retrograde metamorphism across the Dabie orogen. This is in a good agreement with the less high hydrothermal reequilibration temperatures quantified below.

### Theoretical inversion of initial oxygen isotopes of water

As oxygen isotopes of quartz were thermodynamically reequilibrated with alkali feldspar at 140±5^o^C for sample 01HP05 from the Hepeng granitoid pluton (Fig. 3a), a low $\delta{^\text{18}\text{O}}_{\text{W}}^{\text{i}}$ value of −11.01±0.43‰ is theoretically inverted for the open system (Table 1). There is no doubt that this water is affiliated with the ancient meteoric water, which externally infiltrated inwards the studied granitoid during the early Cretaceous. With parameters listed in Table 1, the currently lowered oxygen isotopes of rock-forming minerals are well reproduced and a (W/R)_o_ ratio of 1.10±0.02 is accordingly determined for sample 01HP05 (Figs. S1 and 3a). On the other hand, an evolved meteoric water with the modestly low $\delta{^\text{18}\text{O}}_{\text{W}}^{\text{i}}$ value of −3.82±0.01‰ is theoretically inverted from sample 01HP06 at 230^o^C (Table 1). And its oxygen isotopes of rock-forming minerals were currently lowered with a (W/R)_o_ ratio of 0.94±0.01 by the external infiltration of this evolved meteoric water (Figs. S2 and 3a). It is worthwhile pointing out that a high (W/R)_c_ ratio is systematically quantified if the closed system is adopted (arrowed solid vs. dashed vertical lines in Figs. S1 to S4).

Because a hydrothermal reequilibration temperature of 475±4^o^C was thermodynamically achieved between oxygen isotopes of quartz and alkali feldspar for sample 01SCH02 from the Shangcheng granitoid batholith (Fig. 3b), a high $\delta{^\text{18}\text{O}}_{\text{W}}^{\text{i}}$ value of 6.57±0.05‰ is theoretically inverted from the closed system (Table 1). It is convinced that this high $\delta{^\text{18}\text{O}}_{\text{W}}^{\text{i}}$ value is of an affinity with the magmatic water, which internally derived from the studied granitoid. After hydrothermally reequilibrating processes, oxygen isotopes of rock-forming minerals were currently elevated with a (W/R)_c_ ratio of 1.58±0.07 for sample 01SCH02 (Figs. S3 and 3b).

A moderately high $\delta{^\text{18}\text{O}}_{\text{W}}^{\text{i}}$ value of 4.21±0.04‰ is theoretically inverted at 340^o^C from the gneissic country rock intruded by the Tianzhushan granitoid pluton (i.e., sample 01TZS05 in Fig. 2; Table 1). In principle, oxygen isotopes of rock-forming minerals for sample 01TZS05 could be concurrently elevated either by the internally derived metamorphic water or magmatic water externally infiltrated outwards from the adjacent pluton. Our previous study showed, however, that metamorphic water internally derived from this gneissic country rock itself was too low in $\delta{^\text{18}\text{O}}_{\text{W}}^{\text{i}}$value to concurrently elevate oxygen isotopes of rock-forming minerals observed for sample 01TZS05 (see Refs^20,21,24^). In this regard, the external infiltration of magmatic water outwards from the adjacent Tianzhushan granitoid pluton was whereby proposed for concurrently elevating oxygen isotopes of rock-forming minerals with a (W/R)_o_ ratio of 1.23±0.04 for sample 01TZS05 (Figs. S4 and 3b) observed from the gneissic country rock, which is spatially less than 5 km away from the pluton (see GPS data in Table S1). Geochronologically, this is in a good agreement with the early Cretaceous age of 126±20 Ma available for sample 01TZS05 through zircon U-Pb datings^38^. Compared to oxygen isotopes of rock-forming minerals concurrently elevated by the internally derived magmatic water for sample 01SCH02, the concurrent elevation of oxygen isotopes is more evident for sample 01TZS05 (Figs. 2 and 3). This is consistent with that this evolved magmatic water with a moderately high $\delta{^\text{18}\text{O}}_{\text{W}}^{\text{i}}$ value of 4.21±0.04‰ theoretically inverted above was far from equilibrium with the externally infiltrated gneissic country rock studied wherein.

## Discussion

### The reliability of theoretical inversion for $\delta{^\text{18}\text{O}}_{\text{W}}^{\text{i}}$ value

While the ancient meteoric and magmatic water with endmember and evolved $\delta{^\text{18}\text{O}}_{\text{W}}^{\text{i}}$ values are theoretically inverted from oxygen isotopes of the hydrothermally altered rock-forming minerals in this study (Table 1), their reliability needs to be further assessed. As shown in Eq. (1) through (3) in “Methods” section, both hydrothermal reequilibration temperature and initial oxygen isotopes of constituent minerals are prerequisites in order to theoretically invert the $\delta{^\text{18}\text{O}}_{\text{W}}^{\text{i}}$ value. The hydrothermal reequilibration temperatures are thereby calculated on the basis of oxygen isotopes of quartz-alkali feldspar pair observed for corresponding samples. The initial oxygen isotopes of rock-forming minerals, however, are constrained with the observed oxygen isotopes of the inert zircon at magmatic or metamorphic temperatures. In these regards, the role of temperatures is evaluated to validate ${\updelta}{^\text{18}\text{O}}_{\text{W}}^{\text{i}}$ values theoretically inverted in this study.

**Fig. 4:**
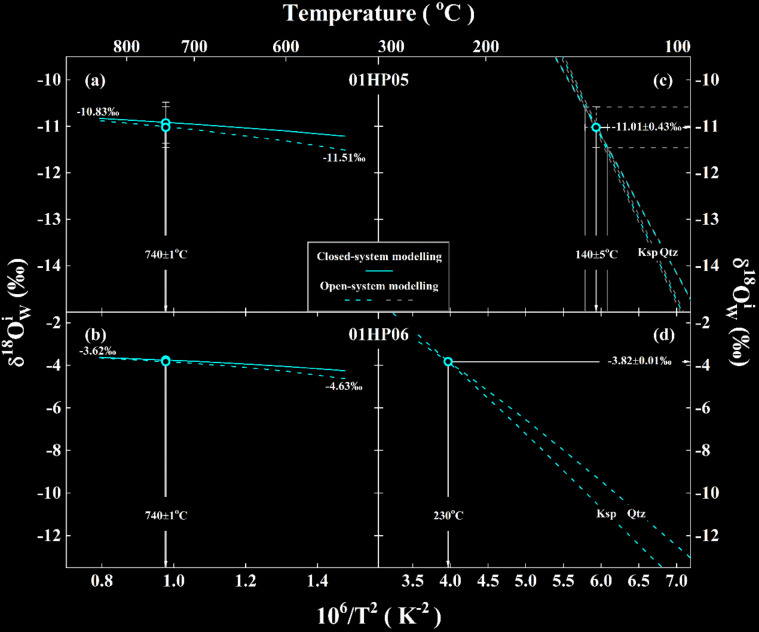
The relationship between temperatures and $\delta{^\text{18}\text{O}}_{\text{W}}^{\text{i}}$ values of ancient meteoric water theoretically inverted from the granitoids of the Hepeng pluton. Arrowed vertical lines illustrate the extreme variation of magmatic (**a**, **b**) and hydrothermal reequilibration temperatures (**c**, **d**) adopted for the studied samples, whereas horizontal ones in (**c**, **d**) demonstrate the extreme variability of $\delta{^\text{18}\text{O}}_{\text{W}}^{\text{i}}$ values. For other details see Table 1 and text.

**Fig. 5:**
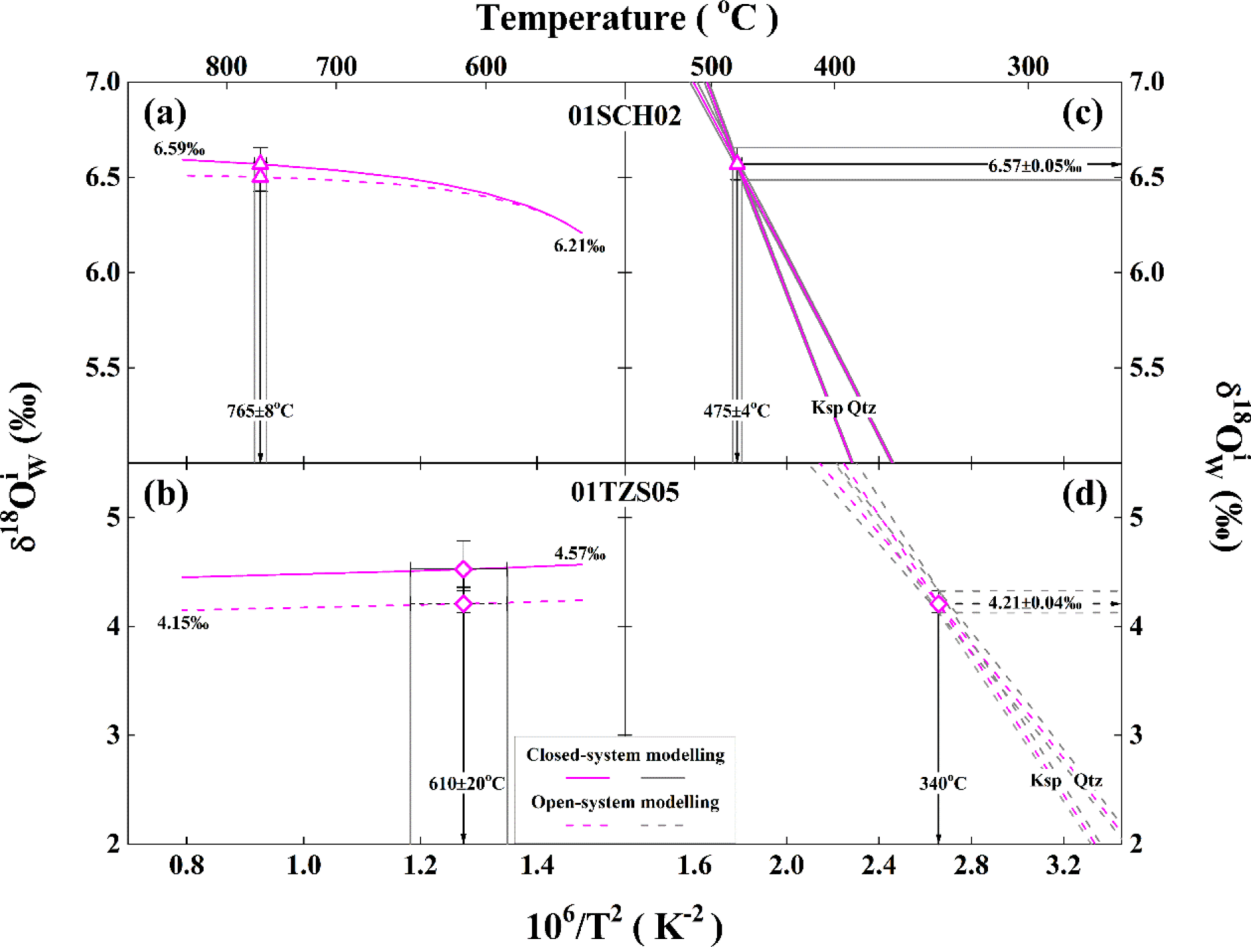
The relationship between temperatures and $\delta{^\text{18}\text{O}}_{\text{W}}^{\text{i}}$ values of magmatic water theoretically inverted from the granitoid (sample 01SCH02) and gneissic country rock (sample 01TZS05), respectively, across the Dabie orogen. Arrowed vertical lines illustrate the extreme variation of magmatic (**a**), metamorphic (**b**) and hydrothermal reequilibration temperatures (**c**, **d**) adopted for the studied samples, whereas those horizontal ones in (**c**, **d**) demonstrate the extreme variability of $\delta{^\text{18}\text{O}}_{\text{W}}^{\text{i}}$ values.

*Construction of the relationship between temperatures and* $\delta{^\text{18}\text{O}}_{\text{W}}^{\text{i}}$ *values.* In order to verify the potential effects of hydrothermal reequilibration temperature and magmatic or metamorphic temperature on theoretical inversion of $\delta{^\text{18}\text{O}}_{\text{W}}^{\text{i}}$ values for the meteoric and magmatic water herein, two strategies are undertaken to deal with these issues separately:

(1) The mean hydrothermal reequilibration temperature is fixed for each studied sample. Because the magmatic temperature up to 765±8^o^C and metamorphic temperature around 610±20^o^C are adopted throughout this study (Table 1; Fig. 3), their extreme variations are arbitrarily set from 550 to 850^o^C. Then, the initial oxygen isotopes of rock-forming minerals are calculated with the observed zircon oxygen isotopes at an assumptively magmatic or metamorphic temperature through Eq. (3). From the low- to high-end, five to seven temperature intervals are usually conducted therein (e.g., 550, 600, 650, …, 850^o^C). Substituting these new initial oxygen isotopes into Eqs. (1) and (2), a hypothetical $\delta{^\text{18}\text{O}}_{\text{W}}^{\text{i}}$ value can be theoretically inverted for the closed system. Similar procedures can be applied to the open system. These results are thus illustrated as labelled curves in Fig. 4a and b and 5a and b.

(2) Owing to the susceptibility of common rock-forming minerals (in particular feldspar) to the hydrothermal alteration, an apparent rather than true hydrothermal reequilibration temperature could be resulted under some situations. In order to test the likely influence of variable hydrothermal reequilibration temperatures on theoretical inversion of ${\updelta}{^\text{18}\text{O}}_{\text{W}}^{\text{i}}$ values, the following procedures are carried out. First, the observed oxygen isotopes of alkali feldspar are fixed for each studied sample. Then, quartz δ^18^O values are reasonably adjusted to either higher or lower ones (five to seven adjustments are usually adequate in most cases). An apparent hydrothermal reequilibration temperature is accordingly calculated with the combination of the observed and adjusted oxygen isotopes. Substituting these values into Eqs. (1) and (2), a hypothetical $\:{\updelta}{^\text{18}\text{O}}_{\text{W}}^{\text{i}}$ value can be theoretically inverted for the closed system. Similar procedures can be applied to the open system and these results are illustrated as curves labelled with Qtz in Figs. 4c and d and 5c and d. When the observed oxygen isotopes of quartz are fixed and alkali feldspar δ^18^O values are adjustable, the resulting curves are labelled with Ksp. It is worthwhile pointing out that the mean magmatic or metamorphic temperatures with extreme variations are adopted for constructing curves with envelops in Figs. 4c and d and 5c and d.

*The impact of magmatic or metamorphic temperatures on* $\delta{^\text{18}\text{O}}_{\text{W}}^{\text{i}}$ *values.* Except for sample 01TZS05, $\delta{^\text{18}\text{O}}_{\text{W}}^{\text{i}}$ values of ancient meteoric and magmatic water theoretically inverted from other samples gradually increase when magmatic temperatures progressively increase (Figs. 4a and b and 5a). Moreover, the subtly higher ${\updelta}{^\text{18}\text{O}}_{\text{W}}^{\text{i}}$ values are systematically inverted for the closed system all over the magmatic temperature range illustrated therein (solid vs. dashed curve in Figs. 4a and b and 5a). The ${\updelta}{^\text{18}\text{O}}_{\text{W}}^{\text{i}}$ values of the evolved magmatic water theoretically inverted from the gneissic country rock, however, steadily decrease with the higher metamorphic temperatures and seem parallel each other between the closed- and open-system (Fig. 5b). With the labelled ${\updelta}{^\text{18}\text{O}}_{\text{W}}^{\text{i}}$ values in Figs. 4a and b and 5a and b, their extreme variability is generally less than ±0.34‰ along with the magmatic or metamorphic temperatures varied for every 100^o^C for studied samples therein. On the other hand, as the actual variation of magmatic or metamorphic temperatures adopted is much more limited (Table 1 and arrowed vertical lines with envelopes in Figs. 4a and b and 5a and b), the corresponding variability of ${\updelta}{^\text{18}\text{O}}_{\text{W}}^{\text{i}}$ values would be much less than the extreme variability of ±0.34‰. For example, the 1SD less than ±0.04‰ is quantified for the ${\updelta}{^\text{18}\text{O}}_{\text{W}}^{\text{i}}$ variability of samples 01HP06 and 01TZS05 (Table 1), which fully depends upon the varied magmatic or metamorphic temperatures. Nevertheless, these suggest that magmatic or metamorphic temperatures adopted in this study can only exert limited influence on the variability of ${\updelta}{^\text{18}\text{O}}_{\text{W}}^{\text{i}}$ values and their reliability is thus guaranteed.

*The effect of hydrothermal reequilibration temperatures on* $\delta{^\text{18}\text{O}}_{\text{W}}^{\text{i}}$ *values.* While the finite impact by magmatic or metamorphic temperatures is evidenced above, the variability of ${\updelta}{^\text{18}\text{O}}_{\text{W}}^{\text{i}}$ values is indeed present (Table 1 and symbol points with error bars in Figs. 4 and 5). In this respect, the effect of hydrothermal reequilibration temperatures is further assessed.

It can be seen that the variability of ${\updelta}{^\text{18}\text{O}}_{\text{W}}^{\text{i}}$ values is more sensitive to hydrothermal reequilibration temperatures. A large range of ${\updelta}{^\text{18}\text{O}}_{\text{W}}^{\text{i}}$ values is theoretically inverted with the variable hypothetical reequilibration temperatures (curves with envelopes in Figs. 4c and d and 5c and d). A cross point, however, appears for each studied sample. This means that both quartz and alkali feldspar oxygen isotopes were virtually reequilibrated with a unique water at the same temperature for individual samples, which just correspond to ${\updelta}{^\text{18}\text{O}}_{\text{W}}^{\text{i}}$ values theoretically inverted with concurrently lowered and/or elevated oxygen isotopes of hydrothermally altered quartz and alkali feldspar (Table 1 and symbol points with error bars in Figs. 4c and d and 5c and d). In these cases, it suggests that thermodynamic reequilibration was attained and/or retained at least for the studied rock-forming minerals and the ancient meteoric and magmatic water with endmember and evolved ${\updelta}{^\text{18}\text{O}}_{\text{W}}^{\text{i}}$ values theoretically inverted therein are validated.

*Assessment of the uncertainties of theoretical inversion.* During theoretical inversion for $\:{\updelta}{^\text{18}\text{O}}_{\text{W}}^{\text{i}}$ values, at least three types of input variables are required. That is, they are the observed oxygen isotopes of rock-forming minerals like $\delta{^\text{18}\text{O}}_{\text{Ksp}}^{\text{f}}$ and ${\updelta}{^\text{18}\text{O}}_{\text{Qtz}}^{\text{f}}$ values, the initial oxygen isotopes of rock-forming minerals like ${\updelta}{^\text{18}\text{O}}_{\text{Ksp}}^{\text{i}}$ and ${\updelta}{^\text{18}\text{O}}_{\text{Qtz}}^{\text{i}}$ values as well as the equilibrium oxygen isotopic fractionation between rock-forming minerals and water like $\:{{{\text{(}}\Delta^{\text{18}}\text{O}}_{\text{W}}^{\text{Ksp}}\text{)}}_{\text{r}}\:$and $\:{{{\text{(}}\Delta^{\text{18}}\text{O}}_{\text{W}}^{\text{Qtz}}\text{)}}_{\text{r}}$values at hydrothermal reequilibration temperatures, respectively, in Eq. (1) through (3). Thus, their direct and/or indirect contributions to the uncertainties of theoretical inversion for ${\updelta}{^\text{18}\text{O}}_{\text{W}}^{\text{i}}$ values are assessed individually below.

For the initial oxygen isotopes of rock-forming minerals, their uncertainties inherit and/or propagate from analytical error of zircon δ^18^O values and variable magmatic or metamorphic temperatures adopted. While the analytical precision of zircon δ^18^O values is the best for sample 01TZS05 among all of available data in this study (±0.01‰ in Table S1), the uncertainties of initial oxygen isotopes of its rock-forming minerals are not correspondingly the smallest because of the large variability of metamorphic temperatures adopted (Table 1, Figs. S4 and 3b). By contrast, owing to the least variation of magmatic temperatures adopted in this study, the uncertainties no more than ±0.02‰ are accordingly constrained for the initial oxygen isotopes of rock-forming minerals for samples 01HP05 and 01HP06 (Table 1, Figs. S1, S2 and 3a). In these cases, it suggests that the uncertainties of initial oxygen isotopes of rock-forming minerals are more dependent upon the variability of magmatic or metamorphic temperatures. Moreover, because the oxygen isotopic fractionation between quartz and zircon is systematically larger than that between alkali feldspar and zircon^28,39–42^, slightly evident uncertainties of the initial oxygen isotopes for quartz secularly appear (Table 1, Figs. S1 through S4 and 3).

The hydrothermal reequilibration temperatures are calculated in terms of the observed oxygen isotopes between quartz and alkali feldspar for studied samples therein. Thus, their uncertainties are mainly regulated by the analytical precision of rock-forming minerals. Because of the highest precision of oxygen isotopes analysed both for quartz and alkali feldspar in this study (±0.01‰ in Table S1), an uncertainty of ±4^o^C is accordingly yielded for the hydrothermal reequilibration temperature of sample 01SCH02 (Table 1; Fig. 5c). As the lack of repetitive measurements for oxygen isotopes of rock-forming minerals for samples 01HP06 and 01TZS05 (Table S1), their 1SD of the hydrothermal reequilibration temperatures is not statistically assigned wherein.

While the extreme variability of ${\updelta}{^\text{18}\text{O}}_{\text{W}}^{\text{i}}$ values theoretically inverted from the closed- and open-system, respectively, is comparable for samples 01HP05, 01HP06 and 01SCH02 (symbol points with error bars in Figs. 4a and b and 5a), a less evident variability is quantified for sample 01TZS05 from the open system (symbol points with error bars in Fig. 5b). These probably implicate that the open system is a more appropriate scenario for the external infiltration of the evolved magmatic water outwards with the gneissic country rock intruded by the Tianzhushan granitoid pluton wherein. The largest 1SD of ±0.43‰ in this study is quantified for ${\updelta}{^\text{18}\text{O}}_{\text{W}}^{\text{i}}$ values of sample 01HP05 although its uncertainty of hydrothermal reequilibration temperature is statistically comparable with that of sample 01SCH02 (±5 vs. ±4^o^C in Table 1; Figs. 4c vs. 5c). In principle, these could intrinsically ascribe to enlarging fractionation of oxygen isotopes between rock-forming minerals and water with declining temperature. For samples 01HP06 and 01TZS05, their variability of ${\updelta}{^\text{18}\text{O}}_{\text{W}}^{\text{i}}$ values seems fundamentally dependent upon the precision of initial oxygen isotopes of rock-forming minerals (Table 1).

An uncertainty of ±0.07 is quantified for the (W/R)_c_ ratio from the closed system for sample 01SCH02 hydrothermally reequilibrated with the internally derived magmatic water (Fig. 3b and solid vertical lines with envelops in Fig. S3). Since open systems are adopted for theoretically inverting $\delta{^\text{18}\text{O}}_{\text{W}}^{\text{i}}$ values of the ancient meteoric and evolved magmatic water (i.e., samples 01HP05, 01HP06 and 01TZS05 in Table 1), limited uncertainties ranging from ±0.01 to ±0.04 are accordingly quantified for their (W/R)_o_ ratios wherein (Fig. 3 and dashed vertical lines with envelops in Figs. S1, S2 and S4).

### Evolution of the ancient meteoric and magmatic water

While meteoric and magmatic water are theoretically inverted from fossil hydrothermal systems and the reliability of respective $\delta{^\text{18}\text{O}}_{\text{W}}^{\text{i}}$ values is validated in the above section, their likely evolution from oxygen isotopic endmembers is further taken into account below.

**Fig. 6:**
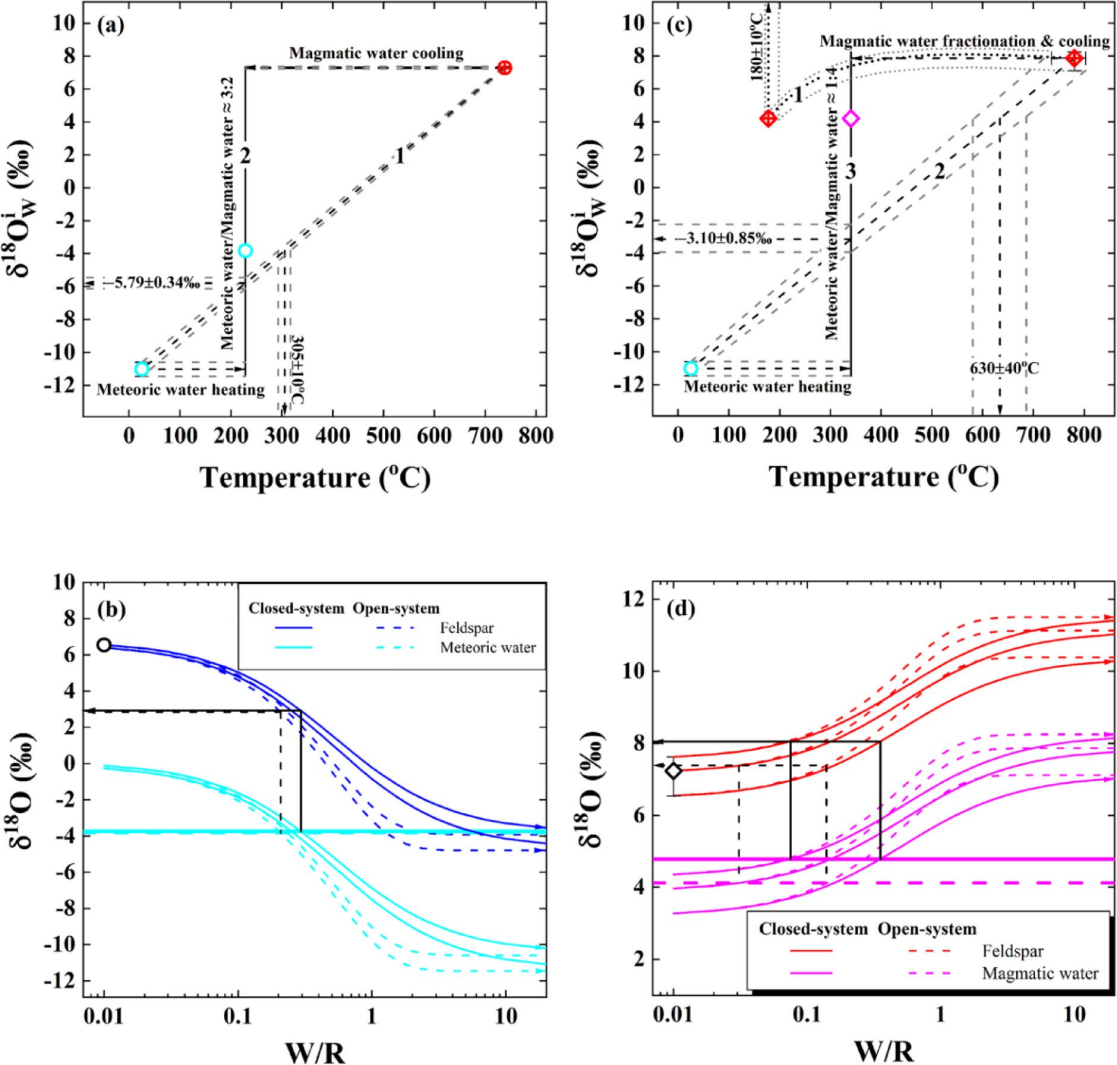
Oxygen isotopic evolution of ancient meteoric and magmatic water. (**a**) Binary mixing pathways between the magmatic and meteoric water for sample 01HP06 with a modestly low $\delta{^\text{18}\text{O}}_{\text{W}}^{\text{i}}$ value of −3.82±0.01‰ (Table 1) under the diabatically (line 1) or isothermal (line 2) conditions, respectively. Oxygen isotopic endmember of the magmatic water (red symbol point with invisible error bars) is calculated with the observed δ^18^O values of zircon at magmatic temperatures and averaged on the scale of the Hepeng granitoid pluton (for other details refer to Table S1 and footnote of Table 1). The endmember of meteoric water is adopted from the $\delta{^\text{18}\text{O}}_{\text{W}}^{\text{i}}$ value theoretically inverted from sample 01HP05 (Table 1), collected from the same pluton. (**b**) Final oxygen isotopes of alkali feldspar and meteoric water after interacting with each other at the hydrothermal reequilibration temperature of 230°C. The initial oxygen isotopes of alkali feldspar are calculated with the observed δ^18^O values of zircon at magmatic temperatures and averaged on the Hepeng pluton scale, whereas those of ancient meteoric water refer to (**a**). Heavy horizontal lines demonstrate the extreme variability of the evolved meteoric water with a modestly low $\delta{^\text{18}\text{O}}_{\text{W}}^{\text{i}}$ value (see sample 01HP06 in Fig. 4b and d). Arrowed lines on the left side exhibit the decreased δ^18^O values of alkali feldspar after coherently exchanging with the meteoric water. (**c**) Curve 1 with envelopes denotes thermodynamic equilibrium fractionation of oxygen isotopes between zircon and magmatic water during the cooling processes, whereas lines 2 and 3 illustrate the binary mixing trajectories between the magmatic and meteoric water for sample 01TZS05 with a moderately high $\delta{^\text{18}\text{O}}_{\text{W}}^{\text{i}}$ value of 4.21±0.04‰ (Table 1) under the diabatic or isothermal conditions, respectively. Oxygen isotopic endmember of the magmatic water (red symbol point with visible error bars) is similarly constrained on the scale of the Tianzhushan granitoid pluton (for other details refer to Table S1, footnote of Table 1 and Ref^24^), whereas that of the ancient meteoric water is adopted from sample 01HP05 (Table 1) and reasonably assumed to be applicable on the orogenic scale. (**d**) Final oxygen isotopes of alkali feldspar and magmatic water after interacting with each other at the hydrothermal reequilibration temperature of 340°C. The initial oxygen isotopes of alkali feldspar are similarly quantified on the Tianzhushan pluton scale, whereas those of magmatic water refer to (**c**). Heavy horizontal lines demonstrate the extreme variability of the evolved magmatic water with a moderately high $\delta{^\text{18}\text{O}}_{\text{W}}^{\text{i}}$ value (see sample 01TZS05 in Fig. 5b and d). Arrowed lines on the left side exhibit the increased δ^18^O values of alkali feldspar after coherently exchanging with the magmatic water.

Since magmatic water is usually enriched in ^18^O (see Ref^43^ and $\delta{^\text{18}\text{O}}_{\text{W}}^{\text{i}}$ values theoretically inverted in Table 1), it could potentially dope oxygen isotopes up to less low values for the externally infiltrated meteoric water. When the two endmembers of meteoric and magmatic water were directly admixed under the diabatic condition, either a high temperature of 305±10°C or a low $\delta{^\text{18}\text{O}}_{\text{W}}^{\text{i}}$ value of −5.79±0.34‰ is accordingly quantified for the mixture therein (arrowed lines with envelopes on the bottom and left sides along with line 1, respectively, in Fig. 6a). By contrast, the modestly low $\delta{^\text{18}\text{O}}_{\text{W}}^{\text{i}}$ value of −3.82±0.01‰ theoretically inverted from the Hepeng granitoid pluton can be reasonably accounted for with a binary mixing under the isothermal condition. That is, a proportion around 39±2 wt% of a cooled but internally derived magmatic water mixed with the heated but externally infiltrated meteoric water at 230^o^C can eventually enable oxygen isotopes of the ancient meteoric water evolved up to the modestly low $\delta{^\text{18}\text{O}}_{\text{W}}^{\text{i}}$ value of −3.82±0.01‰ (line 2 in Fig. 6a).

As what is so called, oxygen isotopes of water and rock can be intrinsically altered each other in the course of water-rock interactions. Thus, the interaction with a host rock could be an alternative for the evolved meteoric water with a modestly low $\delta{^\text{18}\text{O}}_{\text{W}}^{\text{i}}$ value of −3.82±0.01‰. Since alkali feldspar is kinetically more susceptible for exchanging oxygen with water than the resistant quartz and/or inert zircon in hydrothermal processes (for other details see the following section), the evolution of meteoric water is accordingly conducted with alkali feldspar. It can be seen that oxygen isotopes of the meteoric water (cyan curves) were simultaneously increased in coherent response to decreasing oxygen isotopes of alkali feldspar (blue curves) at W/R ratios less than 1 (Fig. 6b). With the extreme variability of the modestly low $\delta{^\text{18}\text{O}}_{\text{W}}^{\text{i}}$ values theoretically inverted from the closed- and open-system (heavy horizontal lines in Fig. 6b), δ^18^O values of alkali feldspar are correspondingly decreased from 2.84 to 2.92‰ (arrowed lines on the left side in Fig. 6b) with W/R ratios from 0.21 to 0.30 at 230°C. While these decreased δ^18^O values of alkali feldspar are not exactly the same as those observed from the Hepeng granitoid pluton (Table S1), they are actually not uncommon across the Dabie orogen (Fig. 2a). Nonetheless, the water-rock interaction prior to the hydrothermal reequilibration of the studied granitoid could be a compelling pathway for the externally infiltrated but evolved meteoric water with a modestly low $\delta{^\text{18}\text{O}}_{\text{W}}^{\text{i}}$ value of −3.82±0.01‰ theoretically inverted therein.

Because of the enlarging fractionation of oxygen isotopes between constituent minerals and water with declining temperature, the moderately high $\delta{^\text{18}\text{O}}_{\text{W}}^{\text{i}}$ value of 4.21±0.04‰ theoretically inverted for magmatic water could in principle evolve from the persistent cooling of magmatic water. High $\delta{^\text{18}\text{O}}_{\text{W}}^{\text{i}}$ values of 7.27±0.59‰, however, were thermodynamically fractionated when the magmatic water diabatically cooled down to the hydrothermal reequilibration temperature of 340°C (curve 1 in Fig. 6c). These high $\delta{^\text{18}\text{O}}_{\text{W}}^{\text{i}}$ values are statistically indistinguishable from those endmembers of magmatic water constrained from the Tianzhushan granitoid pluton (red symbol point with error bars in the upper right of Fig. 6c), and they are apparently too heavy to be appropriate for the evolved magmatic water with a moderately high $\delta{^\text{18}\text{O}}_{\text{W}}^{\text{i}}$ value of 4.21±0.04‰. In order to approach this moderately high $\delta{^\text{18}\text{O}}_{\text{W}}^{\text{i}}$ value, an equilibrium temperature hypothetically cooled down to 180±10^o^C is thermodynamically quantified (arrowed line with envelopes on the top side in Fig. 6c). While this prerequisite equilibrium temperature of 180±10°C seems thermodynamically reasonable, it is doubtlessly inconsistent with the hydrothermal reequilibration temperature of 340°C for theoretically inverting the moderately high $\delta{^\text{18}\text{O}}_{\text{W}}^{\text{i}}$ value of 4.21±0.04‰ therein. Even if the magmatic water could energetically cool down to 180±10^o^C indeed, it had to be thermally looped and reheated up to 340°C again prior to externally infiltrating outwards and hydrothermally reequilibrating with the studied gneissic country rock wherein. Additionally, the hypothetical equilibrium temperature of 180±10°C thermally seems too low to realistically facilitate oxygen exchanging between constituent minerals (in particular zircon whereby) and water in the context of the short-lived magmatic hydrothermal system quantified in the following section. Hence, the magmatic water can persistently cool down even to the room temperature, yet its oxygen isotopes cannot evolve any more via thermodynamic fractionation alone after its host minerals were kinetically blocked and/or frozen for further exchanging oxygen.

The evolved magmatic water with a moderately high $\delta{^\text{18}\text{O}}_{\text{W}}^{\text{i}}$value of 4.21±0.04‰ cannot be satisfactorily diluted by a parcel inflowing of light meteoric water depleted in ^18^O when it directly admixed with the magmatic water under the diabatic condition (line 2 in Fig. 6c). An introduction about 20±2 wt% of the ancient meteoric water, however, can efficiently dilute oxygen isotopes of the magmatic water down to 4.21±0.04‰ by a binary mixing under the isothermal condition of 340°C (line 3 in Fig. 6c). This binary mixing process could first admix thoroughly within the Tianzhushan granitoid pluton and then externally infiltrate outwards with the gneissic country rock adiabatically, or the heated meteoric water isothermally admixed with the cooled magmatic water during their externally infiltrating with the gneissic country rock *en route* wherein. For these two scenarios noted above, the latter one intuitively seems more practical although the first one cannot be exclusively ruled out right now.

An analogous process of water-rock interaction would also mimic the evolved magmatic water with a moderately high $\delta{^\text{18}\text{O}}_{\text{W}}^{\text{i}}$ value of 4.21±0.04‰. In contrast to the interaction between meteoric water and rock discussed above, oxygen isotopes of magmatic water were simultaneously decreased (pink curves) with the coherently increasing oxygen isotopes of alkali feldspar (red curves) after they were hydrothermally interacted each other (Fig. 6d). With the extreme variability of the moderately high $\delta{^\text{18}\text{O}}_{\text{W}}^{\text{i}}$ values theoretically inverted from the closed- and open-system (heavy horizontal lines in Fig. 6d), δ^18^O values of alkali feldspar ranging from 7.39 to 8.05‰ are quantified (arrowed lines on the left side in Fig. 6d) coherently with W/R ratios from 0.03 to 0.35 at 340°C. Since the magmatic water was internally derived from the Tianzhushan granitoid pluton and intrinsically not far from equilibrium with its host minerals, δ^18^O values of alkali feldspar were thus increased less evidently compared to its initial oxygen isotopes. Through this autometasomatism, oxygen isotopes of the magmatic water could first evolve towards the moderately high $\delta{^\text{18}\text{O}}_{\text{W}}^{\text{i}}$ value of 4.21±0.08‰ *in* *situ* when the magmatic hydrothermal system diabatically cooled down to 340°C. Then, the evolved magmatic water adiabatically but externally infiltrated outwards with the gneissic country rock *ex situ* after it had exhumed up to the middle to upper crust.

### Kinetics of oxygen exchange

**Fig. 7:**
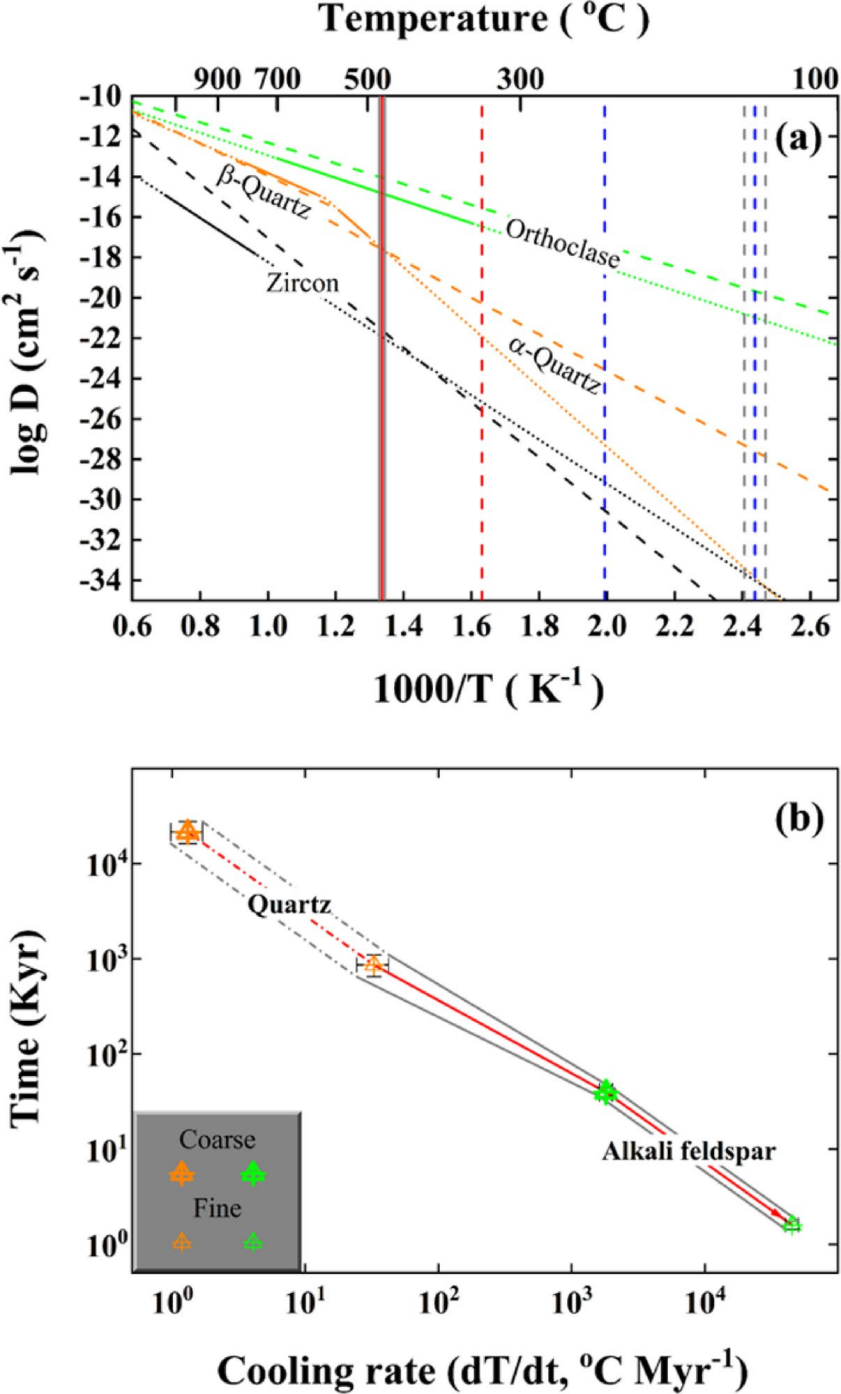
Kinetic modelling of diffusive oxygen exchange. (**a**) Arrhenius plot of oxygen diffusion within minerals under wet conditions. Dashed lines are theoretical calculations for comparison^49^, whereas solid segments with dotted lines are experimental determinations^50–52^. Vertical lines denote hydrothermal reequilibration temperatures between rock-forming minerals and magmatic (red) or meteoric water (blue) in this study (Table 1). (**b**) The relationship between cooling rate and time for diffusive oxygen exchange of rock-forming minerals from the fossil magmatic hydrothermal system kinetically quantified for sample 01SCH02 from the Shangcheng granitoid batholith. Red solid line and dashed/dotted segment with envelopes denote the geologically reasonable and less likely trajectories, respectively. For other details refer to “Method” section, Table 2, Fig. 8 and text.

Diffusion and surface-reaction were previously proposed as fundamental mechanisms for exchanging oxygen between constituent minerals and water from hydrothermal systems^44–48^. Compared to other silicates, the diffusivity of oxygen in zircon is considerably low under similar hydrothermal conditions (Fig. 7). As unrealistic timescales ranging from 205±35 up to 830±140 Myr are kinetically quantified for the diffusive oxygen exchange between zircon and water at 475±4^o^C for sample 01SCH02 (for other details refer to “Methods” section and Table 2), the less resistant rock-forming minerals to hydrothermal alteration are thus considered hereafter.

**Table 2:** Parameters of diffusive oxygen exchange. *From Ref^50^. ^†^Diffusion coefficients parallel to c are adopted after Ref^51^. ^§^From Ref^52^. ^#^Empirically estimated for the medium-grained granitoid. ^∗∗^Hydrothermal reequilibration temperature is from Table 1. ^††^Cooling rate quantified with the procedure described in “Methods” section refers to Fig. 8. Since the log_10_ scale is adopted in Figs. 7 and 8, the absolute value rather than the originally defined one in Eq. (4) with a negative symbol is thus proxied throughout this study instead. ^§§^Time quantified for diffusive oxygen exchange between a spherical mineral and magmatic water refers to “Methods” section, but the Myr is adopted as the unit for zircon herein.

Content	Zircon	α-Quartz	Orthoclase
E_a_ (kJ mole^−1^)	210.2*	284^†^	107.1^§^
D_0_ (cm^2^ s^−1^)	5.5E-08*	190^†^	4.51E-08^§^
Grain radius (a, cm)^#^	0.005 ∼ 0.01	0.05 ∼ 0.25	0.05 ∼ 0.25
Sample 01SCH02 @ T = 475±4^o^C^∗∗^			
Cooling rate (dT/dt, ^o^C Myr^−1^)^††^	/	32.7±7.7 ∼ 1.3±0.3	45,335±4325 ∼ 1810±170
Time (Kyr)^§§^	205±35 ∼ 830±140	860±190 ∼ 21,505±4850	1.6±0.1 ∼ 39.8±3.4

**Fig. 8:**
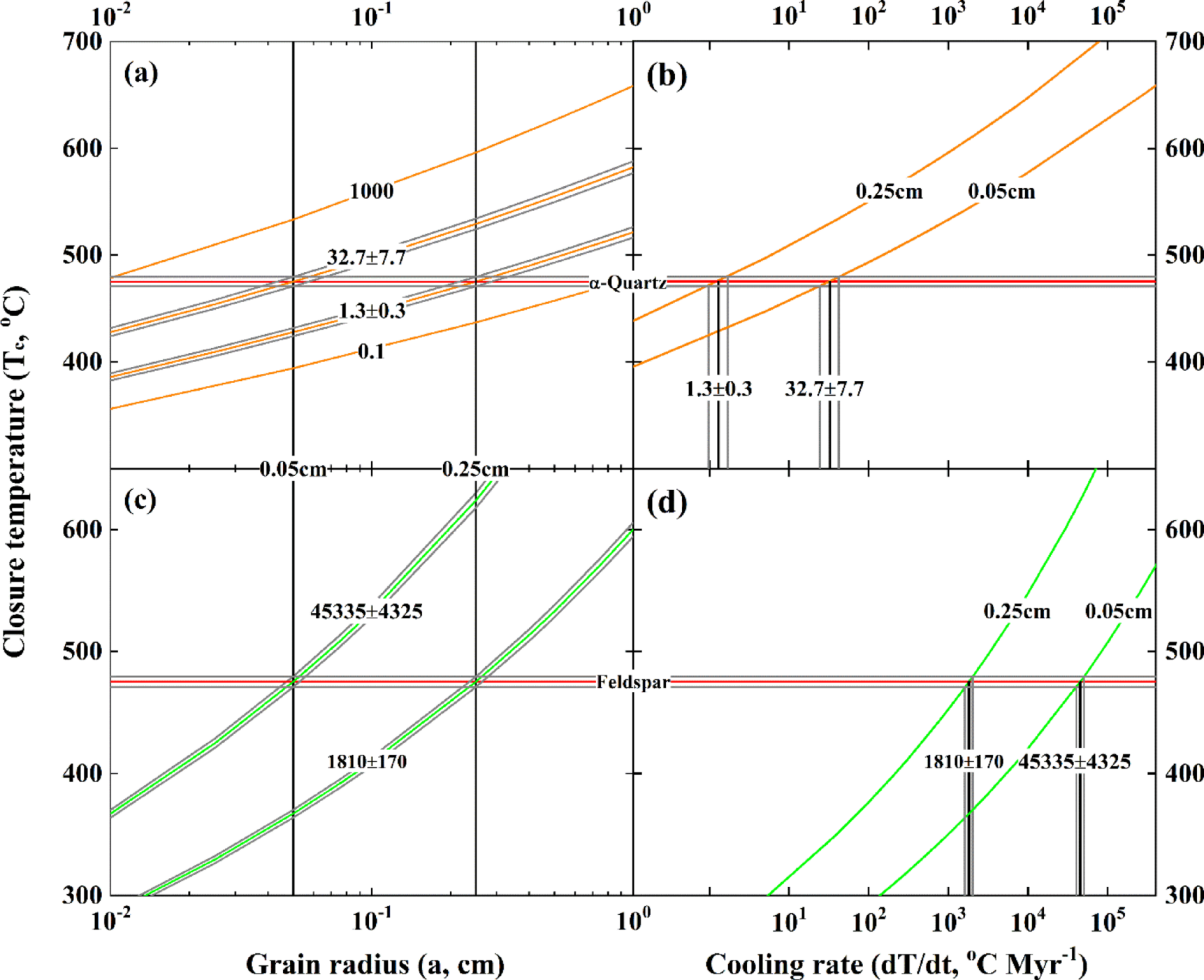
The relationship between closure temperature and grain radius as well as cooling rate of the fossil magmatic hydrothermal system. Red line with envelopes denotes the hydrothermal reequilibration temperature quantified for sample 01SCH02 from the Shangcheng granitoid batholith (Tables 1 and 2). Quartz diffusively exchanging oxygen with the magmatic water is illustrated in (**a**, **b**), whereas alkali feldspar in (**c**, **d**). Vertical lines in (**a**, **c**) denote the grain radius empirically estimated in this study, numbers labelled on the curves are cooling rates with the unit of °C Myr^−1^. Labelled vertical lines with envelopes in (**b**, **d**) denote cooling rates theoretically constrained, whereas the labelled curves are modelled with two grain radii. The conventional log_10_ scale of X axes is adopted for clarity herein.

Based on Eq. (4) described in “Methods” section and parameters listed in Table 2, it can be seen that closure temperatures (i.e., T_c_ values hereafter) gradually increase with the grain size for quartz in the course of diffusive oxygen exchange with the magmatic water at respective cooling rates (labelled curves in Fig. 8a). When the grain size is fixed, high T_c_ values are systematically quantified with the high cooling rates (labelled curves in Fig. 8b). For example, quartz with an extremely coarse-grained size up to around 1 cm is required for the T_c_ value to fit the hydrothermal reequilibration temperature of 475±4^o^C for sample 01SCH02 (red line with envelops throughout Fig. 8) if the geologically slow cooling rate of 0.1°C Myr^−1^ is arbitrarily adopted therein. By contrast, an extremely fine-grained size down to about 0.01 cm is estimated for quartz to diffusively exchange oxygen with the magmatic water with the geologically rapid cooling rate of 1000^o^C Myr^−1^. With the limited grain size empirically estimated in this study (vertical lines in Fig. 8a and labelled curves in Fig. 8b), the cooling rates ranging from 1.3±0.3 to 32.7±7.7°C Myr^−1^ are accordingly quantified for the coarse- and fine-grained quartz, respectively, to diffusively exchange oxygen with the magmatic water at 475±4°C for sample 01SCH02 (Table 2).

On the other hand, similar results are theoretically quantified for the alkali feldspar to diffusively exchange oxygen with the magmatic water. Owing to the rather rapid diffusivity of oxygen for alkali feldspar at 475±4°C for sample 01SCH02 (Fig. 7a), the more evident cooling rates from 1810±170 up to 45,335±4325°C Myr^−1^ are thereby constrained for the coarse- and fine-grained alkali feldspar (labelled curves in Fig. 8c and vertical lines with envelops in Fig. 8d as well as Table 2), respectively. Because of the less sensitivity to temperature (i.e., the relatively flat slope for alkali feldspar in Fig. 7a), the large variability and/or uncertainty is systematically quantified for the cooling rates of alkali feldspar to diffusively exchange oxygen with the magmatic water wherein.

Furthermore, the timescale is kinetically quantified for rock-forming minerals to diffusively exchange oxygen with the magmatic water for sample 01SCH02. A rather long duration of 21,505±4850 Kyr is required for the coarse-grained quartz to diffusively exchange oxygen with the magmatic water with the slow cooling rates quantified in this study (Table 2 and the large orange-colourised symbol demonstrated in Fig. 7b). While this timespan is not theoretically unacceptable, it geologically seems less common for the studied granitoid with a medium-grained texture. In other words, the effective grain radius of quartz would be somehow smaller than the physically coarse-grained size of 0.25 cm empirically estimated therein, and the real cooling rate might be faster than 1.3±0.3^o^C Myr^−1^ theoretically quantified above. A more reasonable time around 860±190 Kyr, however, is accordingly quantified for the fine-grained quartz with a slightly rapid cooling rate to diffusively exchange oxygen with the magmatic water. After the quartz was diffusively blocked and/or isolated from the fossil magmatic hydrothermal system, the coarse- and fine-grained alkali feldspar were then sequentially and quickly blocked with a short duration ranging from 39.8±3.4 to 1.6±0.1 Kyr (Table 2; Fig. 7b). Compared to the sluggish quartz, less variable and/or uncertain timescales are apparently quantified for alkali feldspar with the rapid diffusivity of oxygen. Overall, the lifetime of fossil magmatic hydrothermal system studied herein should be no more than 1 Myr.

Since the hydrothermal reequilibration temperature of 475±4°C is quantified for sample 01SCH02, the hydrothermal reequilibrating between oxygen isotopes of its rock-forming minerals and the magmatic water could temporally take place during the late stage of postmagmatic processes. The shallow emplacement depth within the upper continental crust for the Shangcheng granitoid batholith (Fig. 1) with a typical granitic texture of the medium-grained size also spatially favours the postmagmatic hydrothermal reequilibration. The large size of the heat engine driven by the Shangcheng granitoid batholith, however, can energetically sustain the studied fossil magmatic hydrothermal system alive as long as 1 Myr. Moreover, the cooling rates accelerated from quartz to alkali feldspar for diffusively exchanging oxygen with the magmatic water geologically suggest an enhanced heat loss over time from the fossil magmatic hydrothermal system studied herein. Therefore, the dynamic evolution of the Shangcheng granitoid batholith through continuous and/or periodic erosion-uplift processes would be intuitively inferred.

While a reasonable time around 1.5 Myr is quantitatively constrained for the coarse-grained alkali feldspar to diffusively exchange oxygen with the evolved magmatic water at 340°C, an unrealistic duration over 19,000 Myr is thereby quantified even for the fine-grained quartz of sample 01TZS05. Given that hydrothermal reequilibration temperatures from 140±5 to 230°C are quantified for samples 01HP05 and 01HP06 (Table 1), they also seem less likely for hydrothermally reequilibrating with ancient meteoric water via diffusion dominated processes due to the systematically slowing down of oxygen diffusion rates for alkali feldspar and in particular quartz at these temperature intervals (see the intersections between the blue vertical lines and green as well as orange curves in Fig. 7a). Under these instances, the mechanism of surfacereaction oxygen exchange is taken into account below.

Compared to diffusive processes, oxygen exchange rates of the surface-reaction between constituent minerals and water are several orders of magnitude high (see Refs^53,54^ and r values in Table 3). Since hydrothermal reequilibrations were achieved and/or reproduced between oxygen isotopes of rock-forming minerals and water for the studied granitoids and gneissic country rock (i.e., samples 01HP05, 01HP06 and 01TZS05 in Figs. S1, S2, S4 and labelled data points in Fig. 3), these probably suggest that the surface-reaction instead of volume diffusion actually governed the oxygen exchange wherein. Mechanisms of the surface-reaction such as dissolution, reprecipitation and exchange along micro-fractures and/or within networks were proposed to account for the variation of oxygen isotopes for quartz and/or alkali feldspar during hydrothermal processes^53–57^.

**Table 3:** Parameters of surface-reaction oxygen exchange. *Hydrothermal reequilibration temperatures are from Table 1. ^†^Closed system (W/R)_c_ ratios refer to Figs. S1, S2 and S4. ^§^Mole fraction of mineral oxygen hydrothermally reequilibrated with water. ^#^Mineral density from Ref^58^. **Rate constant after Refs^53,54^. ^††^Grain radius empirically estimated for the medium-grained granitoid and gneissic country rock. ^§§^Time required for attaining 99% oxygen exchange (i.e., F value in the following equation) between a spherical mineral and water. As previously formulated for the closed system^53,54^, $\:\text{t}=\text{}\frac{\text{}\text-{\text{ln}(1}\text{}-\text{F)}\text{}{\text{(W/R)}}_{\text{c}}\text{}{\text{X}}_{\text{s}}\text{}\text{a}\text{}\rho}{\text{3}\text{}\left[\text{1}+\text{}{\text{(W/R)}}_{\text{c}}\right]\text{}\text{r}\text{}{\text{10}}^{\text{-4}}}$, where all variables are listed within Table 3. Since the formulation for the open system is still unavailable up to now, the time quantified herein is more or less an approximation for the true time required by the open system.

Sample number	T (^o^C)*	(W/R)_c_^†^	Alkali feldspar					Quartz				
			Xs^§^	ρ^#^ (g cm^−3^)	log r** (moles O m^−2^ s^−1^)	a^††^ (cm)	t^§§^ (Kyr)	Xs^§^	ρ^#^ (g cm^−3^)	log r** (moles O m^−2^ s^−1^)	a^††^ (cm)	t^§§^ (Kyr)
**Granitoid**				2.56					2.66			
01HP05	140±5	4.49±0.15	0.182±0.005		−8.71±0.08	0.05	5±1	0.182±0.005		−10.18±0.10	0.05	145±20
						0.25	20±3				0.25	730±105
01HP06	230	3.01±0.01	0.250±0.001		−7.86	0.05	0.8±0.0	0.250±0.001		−9.13	0.05	16.2±0.1
						0.25	4.2±0.0				0.25	80.9±0.2
**Gneiss**												
01TZS05	340	2.91±0.32	0.258±0.021		−7.17	0.05	0.2±0.0	0.258±0.021		−8.27	0.05	2.3±0.1
						0.25	0.9±0.0				0.25	11.4±0.6

**Fig. 9:**
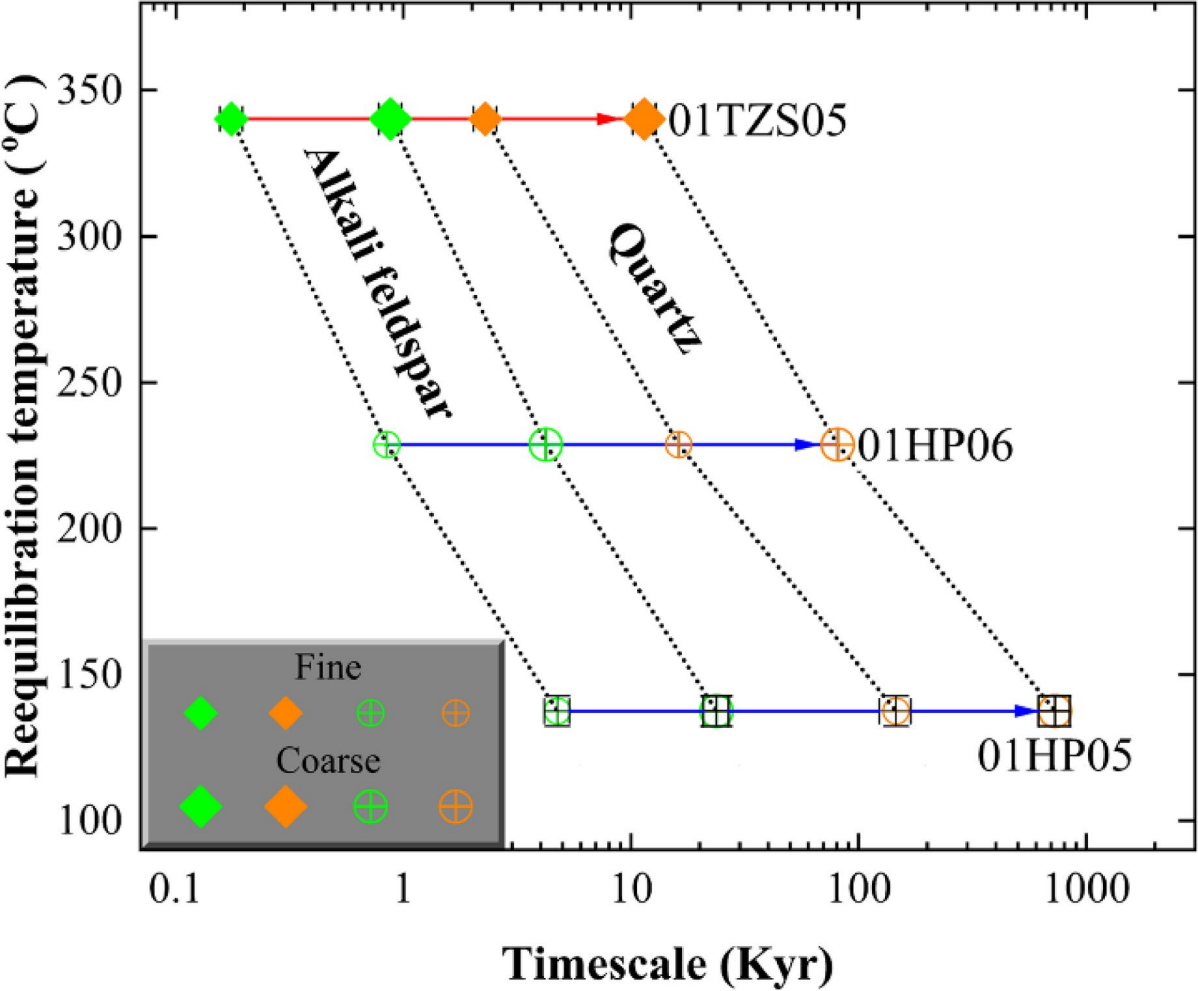
Kinetic modelling of surface-reaction oxygen exchange. Symbol points denote mean values of rock-forming minerals with fine- or coarse-grained size sequentially reequilibrated with the evolved magmatic (sample 01TZS05) or ancient meteoric water (samples 01HP05 and 01HP05), respectively, in the closed hydrothermal systems. For other details refer to Table 3 and text.

On the basis of parameters listed in Table 3 and the corresponding formulation wherein, the time for attaining 99% oxygen exchange between rock-forming minerals and water is accordingly calculated. Since the oxygen exchange rate between alkali feldspar and water is more rapid than that between quartz and water^53,54^, hydrothermal reequilibrations were readily approached for the fine-grained alkali feldspar at the temperature intervals throughout this study (Table 3; Fig. 9). Then, a longer timescale is systematically quantified for the coarse-grained quartz.

Durations from 0.2±0.0 to 11.4±0.6 Kyr are thereby quantified for alkali feldspar and quartz to sequentially exchange oxygen with the evolved magmatic water at 340°C for sample 01TZS05 from the gneissic country rock (Table 3 and red arrowed line in Fig. 9). In other words, the wholesale lifetime of the studied fossil magmatic hydrothermal system could be less than 12 Kyr. This is in a good agreement with the limited size of the Tianzhushan granitoid pluton (Fig. 1), which cannot energetically maintain its hydrothermal system alive sufficiently long. Geologically, this magmatic hydrothermal system could spatiotemporally operate during the late stage of the adjacent Tianzhushan granitic magmatism and/or be sustained by the upper (outer) portion of this pluton prior to externally infiltrating outwards with the gneissic country rock wherein.

Time intervals from 0.8±0.0 to 730±105 Kyr are quantified for the surface-reaction oxygen exchanging between the externally infiltrated meteoric water and rock-forming minerals of the studied granitoids (i.e., samples 01HP05 and 01HP06 in Table 3 and blue arrowed lines in Fig. 9). Since the hydrothermal reequilibration temperatures are overall less than 230°C wherein, these fossil meteoric hydrothermal systems should temporally develop at the end of the early Cretaceous postcollisional magmatism. The external infiltration by a cold meteoric water could thermally enhance the cooling of these fossil hydrothermal systems. The upper continental crust settings intruded by the Hepeng granitoid pluton (Fig. 1) were spatially in accordance with the promoted cooling of the studied hydrothermal systems wherein. The intermediate size of the Hepeng granitoid pluton itself, however, can energetically sustain these fossil meteoric hydrothermal systems alive up to 0.8 Myr.

## Methods

### Geologic setting and sampling

The Dabie-Sulu orogens, as previously summarised by our works^20–24^, have been characterised by the largest occurrence of the microdiamond- and/or coesite-bearing UHP metamorphic rocks around the world^59–63^. The eclogite facies rocks with Triassic ages of 200 to 240 Ma were dated by distinct geochronometers and their cooling histories during exhumation processes were accordingly quantified^32–38^. In addition, the ultrahigh ε_Nd_(t) values up to + 264 ever measured for eclogites^64 ^and zircon with the lowest ever reported δ^18^O values down to about − 11‰ were found wherein^65–67^.

In contrast to the sporadically outcropped lenses or blocks of UHP eclogites, composite plutons and batholiths of the early Cretaceous postcollisional granitoid are predominant igneous rocks although a number of coeval small mafic to ultramafic plutons have been documented across the Dabie orogen^25,26,68–81^. Most plutons and batholiths were geochronologically dated with the early Cretaceous ages ranging from 125 to 135 Ma via zircon U-Pb techniques. The upper intercept ages of Neoproterozoic for available zircon U-Pb datings indicate their affinities with the South China Block. An old enriched source(s) is petrogenetically linked to the early Cretaceous postcollisional igneous rocks with zircon Hf and whole-rock Nd-Sr isotopes.

The studied granitoids and their gneissic country rocks spatially occupy the northern and eastern lithotectonic units of the Dabie orogen (Fig. 1). From north to south, the Shangcheng batholith (SCH) and Hepeng pluton (HP) outcrop in the utmost northern and eastern tips of the Northern Huaiyang volcanic-sedimentary belt, whereas the Tianzhushan pluton (TZS) is adjacent to the Central Dabie UHP metamorphic belt. The intrusive contact between granitoids and gneissic country rocks was unambiguously observed in the field. For comparison, two gneisses from the Sidaohe without being intruded by granitoids in the Hong’an Block are also studied herein.

Fresh and/or less weathered medium-grained granitoids and gneisses were collected from quarries and/or along road cuttings. Petrographically, quartz, feldspar, biotite and sometimes amphibole are common constituent minerals, and accessory minerals include zircon and magnetite.

### Analysis of oxygen isotopes

Zircon, quartz and alkali feldspar were separated and concentrated from whole-rocks through conventional crushing, gravimetric, heavy liquid and magnetic techniques. The separated zircons were then sequentially treated with concentrated HCl, HNO_3_ and HF acids under room conditions overnight to eliminate metamict zircons and other impurities. The purity of mineral separates is generally better than 98% with optical microscope examination.

Oxygen isotopes were analysed with the laser fluorination online techniques^82,83^, and an air-lock chamber was employed to avoid the “cross-talk” of reactive alkali feldspar^84^. The conventional δ^18^O notation in permil (‰) relative to the Vienna Standard Mean Ocean Water (VSMOW) is reported in Table S1.

The garnet standard, UWG-2, was routinely analysed to control the quality of δ^18^O analyses. For 15 analytical days over three months, the daily average of measured δ^18^O values of UWG-2 varied from 5.54 to 5.89‰, and the daily analytical precision is better than ±0.11‰. Raw δ^18^O values of mineral separates were accordingly corrected in terms of the accepted UWG-2 value of 5.80‰. The international standard, NBS 28 quartz, was analysed and the corrected δ^18^O values for NBS 28 are from 9.31 to 9.69‰ during the course of this study.

The reproducibility of fresh crystalline zircon δ^18^O analyses is excellent throughout this study. The 1SD of most duplicate measurements with one triplicate is less than ±0.05‰ (Table S1), which is within the maximum routine analytical errors demonstrated by daily UWG-2 garnet standard measurements.

### Theoretical inversion of $\delta{^\text{18}\text{O}}_{\text{W}}^{\text{i}}$ value

The $\delta{^\text{18}\text{O}}_{\text{W}}^{\text{i}}$ value can be theoretically inverted from constituent minerals achieved thermodynamic reequilibration with water^20–24^. For the closed system, two equations were derived for major rock-forming minerals such as alkali feldspar (Ksp) and quartz (Qtz), respectively:

1 $$\delta{^\text{18}\text{O}}_{\text{Ksp}}^{\text{f}}\text{}=\frac{\delta{^\text{18}\text{O}}_{\text{Ksp}}^{\text{i}}+\text{}\left[{\delta{^\text{18}\text{O}}_{\text{W}}^{\text{i}}\text{}+{{\text{(}\text{}}\Delta^{\text{18}}\text{O}}_{\text{W}}^{\text{Ksp}}\text{)}}_{\text{r}}\right]\text{}\cdot{\left(\text{W}/\text{R}\right)}_{\text{c}}\cdot\text{}\left({\text{n}}_{\text{W}}^{\text{O}}/{\text{n}}_{\text{Ksp}}^{\text{O}}\right)}{\text{1}+\text{}{\left(\text{W}/\text{R}\right)}_{\text{c}}\cdot\text{}\left({\text{n}}_{\text{W}}^{\text{O}}/{\text{n}}_{\text{Ksp}}^{\text{O}}\right)}$$

2 $$\delta{^\text{18}\text{O}}_{\text{Qtz}}^{\text{f}}\text{}=\frac{\delta{^\text{18}\text{O}}_{\text{Qtz}}^{\text{i}}+\text{}\left[{\delta{^\text{18}\text{O}}_{\text{W}}^{\text{i}}\text{}{{\text{+(}\text{}}\Delta^{\text{18}}\text{O}}_{\text{W}}^{\text{Qtz}}\text{)}}_{\text{r}}\right]\cdot\text{}{\left(\text{W}/\text{R}\right)}_{\text{c}}\cdot\text{}\left({\text{n}}_{\text{W}}^{\text{O}}/{\text{n}}_{\text{Qtz}}^{\text{O}}\right)}{\text{1}+\text{}{\left(\text{W}/\text{R}\right)}_{\text{c}}\cdot\text{}\left({\text{n}}_{\text{W}}^{\text{O}}/{\text{n}}_{\text{Qtz}}^{\text{O}}\right)}$$

 

3 $$\text {and}\,\delta{^\text{18}\text{O}}_{\text{Ksp}}^{\text{i}}=\text{}\delta{^\text{18}\text{O}}_{\text{Zrc}}^{\text{i}}\text{}{{{\text+{(}\text{}}\Delta^{\text{18}}\text{O}}_{\text{Zrc}}^{\text{Ksp}}\text{)}}_{\text{m}}\,\text {or}\,\delta{^\text{18}\text{O}}_{\text{Qtz}}^{\text{i}}=\text{}\delta{^\text{18}\text{O}}_{\text{Zrc}}^{\text{i}}\text{}{{{\text+{(}\text{}}\Delta^{^\text{18}}\text{O}}_{\text{Zrc}}^{\text{Qtz}}\text{)}}_{\text{m}}\text{}\text{}\text{}$$

where $\delta{^\text{18}\text{O}}_{\text{Ksp}}^{\text{f}}$ and $\delta{^\text{18}\text{O}}_{\text{Qtz}}^{\text{f}}$ denote final values observed for specified minerals after being hydrothermally altered; $\delta{^\text{18}\text{O}}_{\text{Ksp}}^{\text{i}}$ and $\delta{^\text{18}\text{O}}_{\text{Qtz}}^{\text{i}}$ denote initial values calculated by Eq. (3) with the observed zircon (Zrc) oxygen isotopes as the $\delta{^\text{18}\text{O}}_{\text{Zrc}}^{\text{i}}$ value at the magmatic or metamorphic temperature through the equilibrium fractionation between the specified mineral and zircon ($\:{{{\text{i.e.,(}\text{}}\Delta^{\text{18}}\text{O}}_{\text{Zrc}}^{\text{Ksp}}\text{)}}_{\text{m}}\text{or}\:{{{\text{(}\text{}}\Delta^{\text{18}}\text{O}}_{\text{Zrc}}^{\text{Qtz}}\text{)}}_{\text{m}}\text{value}$); and $\:{{{\text{(}\text{}}\Delta^{\text{18}}\text{O}}_{\text{W}}^{\text{Ksp}}\text{)}}_{\text{r}}\:$and $\:{{{\text{(}\text{}}\Delta^{\text{18}}\text{O}}_{\text{W}}^{\text{Qtz}}\text{)}}_{\text{r}}$ denote the equilibrium fractionation between the specified mineral and water, which can be calculated with the hydrothermal reequilibration temperature. Moreover, $\:{\text{n}}_{\text{W}}^{\text{O}}/{\text{n}}_{\text{Ksp}}^{\text{O}}\text{and}\,{\text{n}}_{\text{W}}^{\text{O}}/{\text{n}}_{\text{Qtz}}^{\text{O}}$ ratios denote exchangeable oxygen content between water and the specified mineral, which are actually constants (last row in Table 1). In these circumstances, both $\delta{^\text{18}\text{O}}_{\text{W}}^{\text{i}}$ value and $\:{\left(\text{W}/\text{R}\right)}_{\text{c}}$ ratio can thus be uniquely solved by combining Eqs. (1) and (2).

Theoretically calculated oxygen isotopic fractionations at temperatures ranging from 0 to 1200^o^C are adopted throughout this study in order to be self-consistent^28^. Because the discrepancy between theoretical calculation and experimental calibration or empirical estimation is not remarkable for oxygen isotopic fractionations amongst the studied constituent minerals^39–42^, this will not considerably influence results of our study herein.

A similar inverse procedure can be applied to the open system. Due to the term of natural logarithmic or exponential function (i.e., (W/R)_o_=ln[(W/R)_c_+1]), an analytical expression cannot be obtained. Under this case, the numerical reiteration with a goal precision of at least ±0.0001 is conducted for theoretically inverting the $\delta{^\text{18}\text{O}}_{\text{W}}^{\text{i}}$ value and (W/R)_o_ ratio wherein.

### Diffusion, cooling rate and lifetime of fossil hydrothermal system

As an elementary process of the atomistic migration, chemical diffusion undoubtedly plays one of essential roles for exchanging oxygen between constituent minerals and water during high-temperature and -pressure geologic events. While fossil hydrothermal systems globally developed within various continental and oceanic settings, their history of thermal evolution is still not a well resolved issue. The main reason is lacking candidate minerals reliably dated, formed during hydrothermal alterations analogously alike authigenic minerals in sedimentary processes. In contrast to the scarcity of radionuclides enriched within hydrothermally altered minerals (e.g., the mostly common occurrence of quartz), diffusive oxygen exchange is more popular and readily achievable between constituent minerals and water. Additionally, it has been well known that the diffusion is thermally activated (i.e., the simplified Arrhenius relationship of $\:\text{D}={\text{D}}_{\text{0}}{\text{e}}^{{\text{E}}_{\text{a}}/\text{RT}}$ shown in Fig. 7a at constant pressures). Thereby, thermal history of fossil hydrothermal systems can be in principle characterised by the corresponding hydrothermal reequilibration temperatures. If the hydrothermal reequilibration was indeed predominated by the diffusive oxygen exchange between constituent minerals and water, the hydrothermal reequilibration temperature quantified in this study would be actually equivalent to the well-established closure temperature.

As originally formulated by Dodson^85^, the bulk T_c_ value is fundamentally satisfied with the following relationship for the chemical diffusion dominant cooling systems:

4 $$\:\:{\text{T}}_{\text{c}}=\frac{{\text{E}}_{\text{a}}/\text{R}}{\text{l}\text{n}\left[-\frac{\text{A}\text{R}{\text{T}}_{\text{c}}^{2}\left({\text{D}}_{0}/{\text{a}}^{2}\right)}{{\text{E}}_{\text{a}}\left(\text{d}\text{T}/\text{d}\text{t}\right)}\right]}$$

where E_a_ is the activation energy of a certain diffusing species of geological interest, R is the gas constant, A is the geometrical factor with a value of 55 for spherical minerals studied therein, D_0_ is the preexponential factor, a is the grain radius and dT/dt is the cooling rate, respectively. Thus, T_c_ value can be numerically reiterated when all parameters are known and/or reasonably assumed wherein.

Amongst all variables in Eq. (4), the diffusion coefficients (i.e., E_a_ and D_0_) of a diffusant (oxygen thereafter) within minerals are available from experimental determination, theoretical calculation and empirical estimation (for other details refer to Fig. 7a; Table 2). The grain radius (i.e., a in Eq. (4)) of a specified mineral can be manually (or automatically in recent years) measured with optical and/or electron microscope or empirically estimated in the field. The cooling rate (i.e., dT/dt in Eq. (4)), however, is the most problematic and less rigorous one in most previous applications. For example, the geologically plausible cooling rates ranging from 0.1 to 1000^o^C Myr^−1 ^were arbitrarily adopted with at least four orders of magnitude of variability^4–6^. Additionally, because most T_c_values were geochronologically oriented, an assumption of linear cooling rate with age for the dated mineral was applicable to young samples (cf., Eqs. (8) and (9) in Ref^86^). Furthermore, the cooling rate of a rock is seriously enigmatic. Except for early Rb-Sr and/or K-Ar isochron datings with whole-rocks, modern geochronological studies have been undertaken with minerals or even within mineral scale by *in situ* approaches recently^87–90^. Geothermometry with oxygen isotopes has been universally conducted with mineral pairs since its birth^10–13^. When a cooling rate was assumed or adopted for a rock (or sample in fact wherein), it was implicitly applied to each constituent mineral with the same cooling rate by most previous studies. The justice of these assumptions absolutely necessitates an independent examination and/or quantitative quantification.

Substituting T_c_ value with the hydrothermal reequilibration temperature quantified in this study into Eq. (4), however, a unique cooling rate for the corresponding mineral can be theoretically inverted individually (see Fig. 8; Table 2). Moreover, since the model of Dt/a^2^=0.03 is mathematically held for a spherical crystal, the timescale of diffusive oxygen exchange between hydrothermally altered minerals and water can be further quantified. In this regard, the cooling rate and lifetime of fossil hydrothermal systems can be theoretically constrained therein (cf., Table 2; Figs. 7b and 8).

## Electronic supplementary material

Below is the link to the electronic supplementary material.

Supplementary Material 1

## Data Availability

The authors declare that all relevant data are available within the article and its Supplementary Information Files.
