# Supplementary material for: Evolution of ancient hydrothermal fluids theoretically inverted with initial oxygen isotopes of water
Source: Sci Rep. 2025 May 6;15:15803. doi: 10.1038/s41598-025-99653-x (PMC12056045; doi:10.1038/s41598-025-99653-x)
Supplement: Supplementary file 1 — Supplementary Material 1 [file 41598_2025_99653_MOESM1_ESM.pdf]

Supplementary Information for

# Evolution of ancient hydrothermal fluids theoretically inverted with initial oxygen isotopes of water

**Chun-Sheng Wei**✉ **& Zi-Fu Zhao**

CAS Key Laboratory of Crust-Mantle Materials and Environments, School of Earth and Space Sciences, University of Science and Technology of China, Hefei 230026, China. ✉email: wchs@ustc.edu.cn

**This PDF file includes:**

Figs. S1 to S4

Table S1

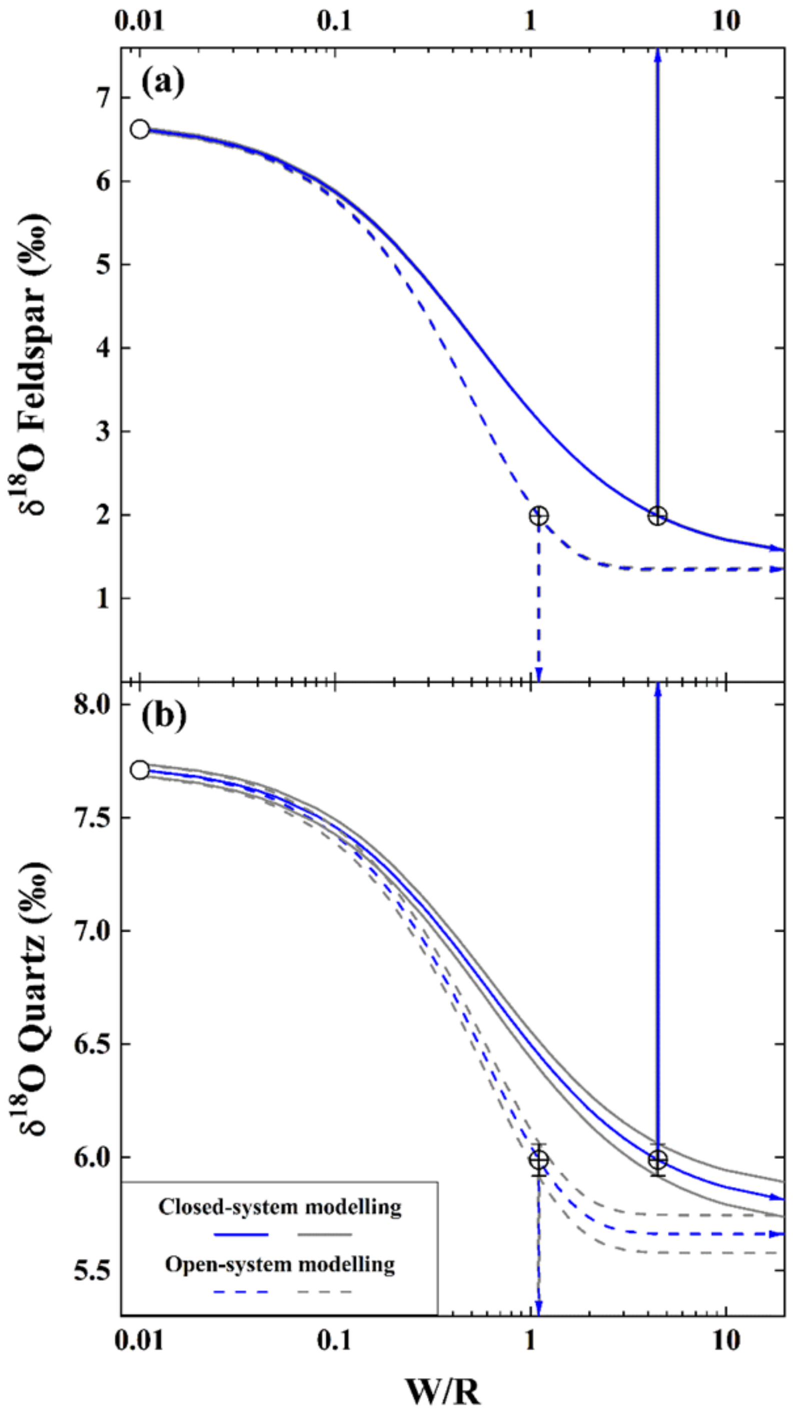


**Figure S1.** The concurrently lowered oxygen isotopes with W/R ratios for sample 01HP05 by the external infiltration of ancient meteoric water inwards the early Cretaceous postcollisional Hepeng granitoid pluton. (**a**) Alkali feldspar. (**b**) Quartz. Due to the limited variability of the observed and initial oxygen isotopes, the envelopes are almost invisible for alkali feldspar in (**a**). Arrowed vertical lines illustrate W/R ratios required to reproduce the observed $\delta^{18}O$ values. Note that $\log_{10}$ scale of X axes and different scales of Y axes in (**a**) and (**b**) are adopted for clarity. For other details refer to Figs. 2 and 3.

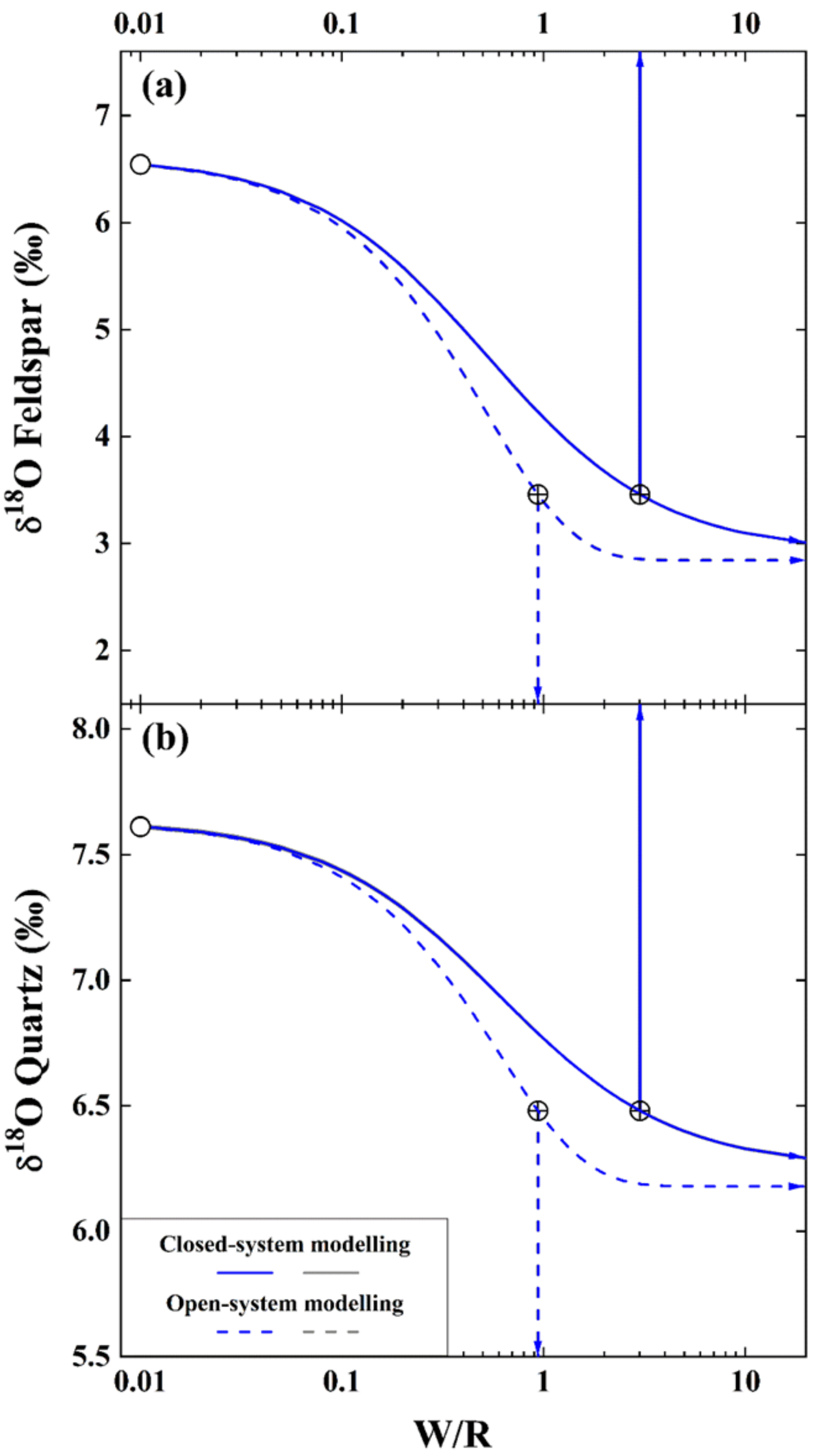


**Figure S2.** The concurrently lowered oxygen isotopes with W/R ratios for sample 01HP06 by the external infiltration of ancient meteoric water inwards the early Cretaceous postcollisional Hepeng granitoid pluton. Due to the limited variability of the observed and initial oxygen isotopes, the envelopes are almost invisible herein. For other details refer to Fig. S1.

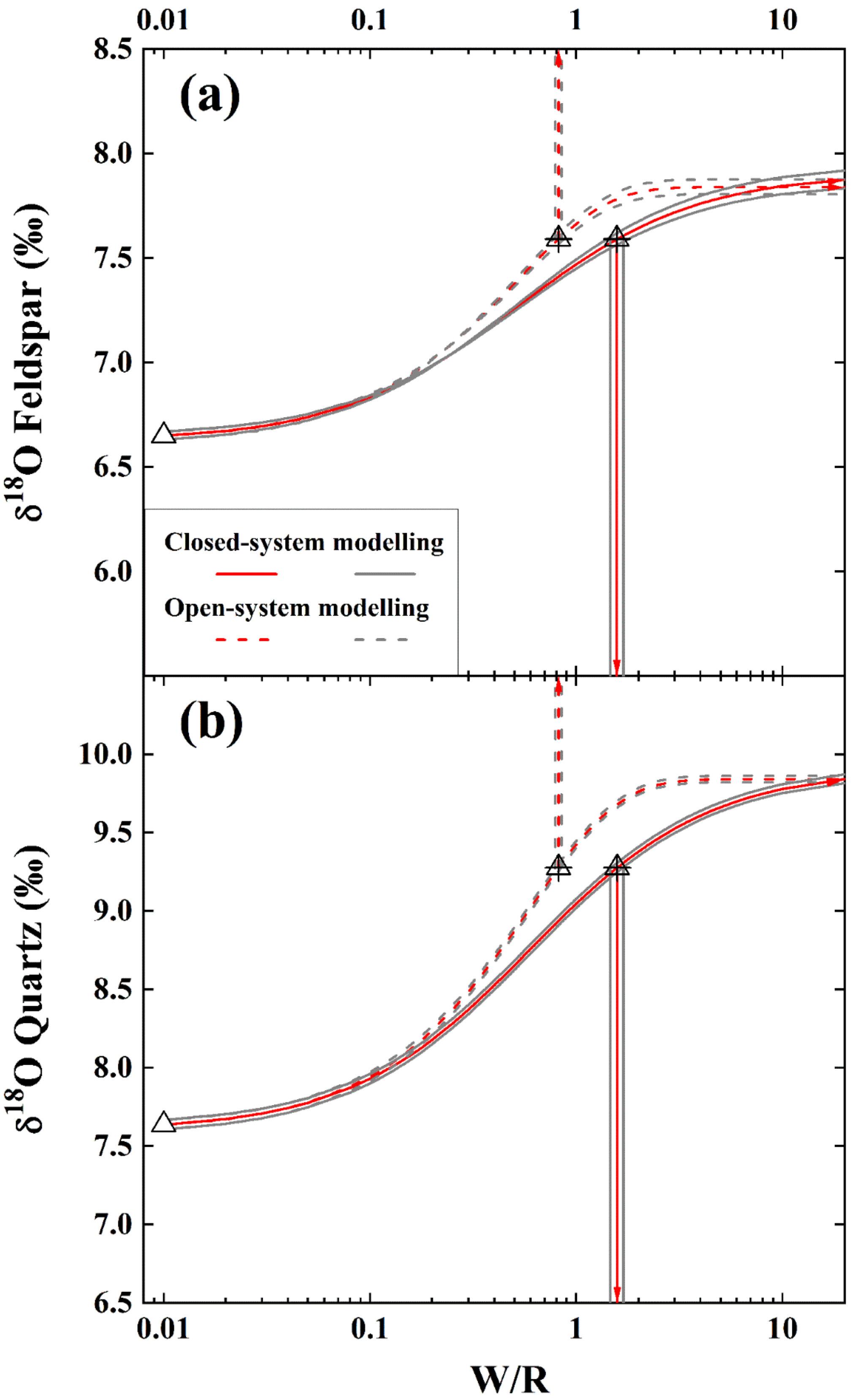


**Figure S3.** The concurrently elevated oxygen isotopes with W/R ratios for sample 01SCH02 by the magmatic water internally derived from the Shangcheng granitoid batholith. For other details refer to Fig. S1.

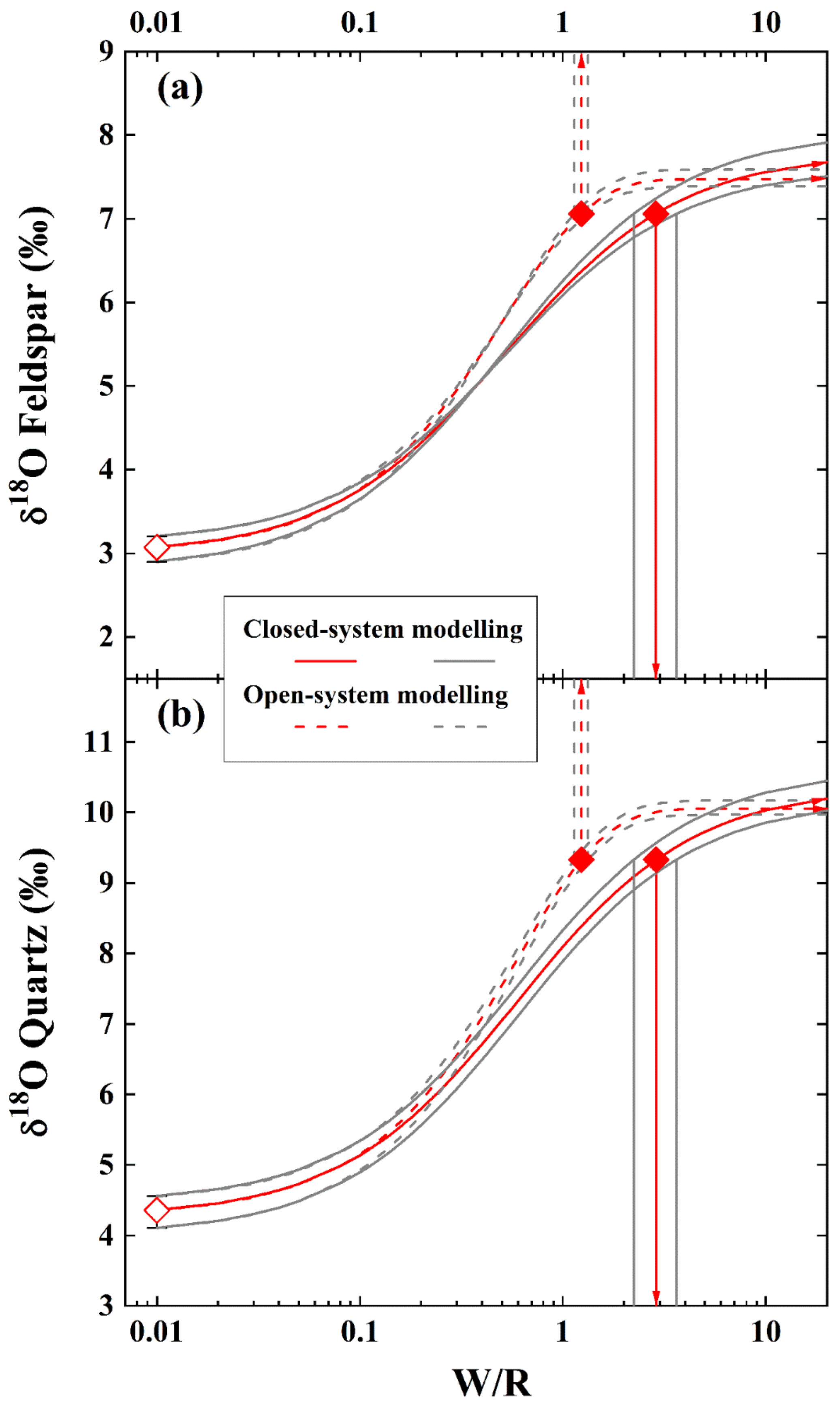


**Figure S4.** The concurrently elevated oxygen isotopes with W/R ratios for sample 01TZS05 by the external infiltration of magmatic water inwards the gneissic country rock intruded by the Tianzhushan granitoid pluton. For other details refer to Fig. S1.

| Sample number | δ18O Zircon (‰) | | | δ18O Quartz (‰) | | | δ18O Alkali feldspar (‰) | | | GPS data |
|---|---|---|---|---|---|---|---|---|---|---|
| | Measured | Ave | 1SD | Measured | Ave | 1SD | Measured | Ave | 1SD | |
| Hepeng pluton (HP) | | | | | | | | | | |
| Granitoid | | | | | | | | | | |
| 01HP04† | 4.55, 4.54 | 4.55 | 0.01 | 7.69 | 7.69 | / | 6.42 | 6.42 | / | 31º13′37″, 116º45′25″ |
| 01HP05† | 4.64, 4.60 | 4.62 | 0.03 | 5.92, 6.06 | 5.99 | 0.10 | 1.99 | 1.99 | / | 31º12′42″, 116º47′28″ |
| 01HP06 | 4.51 | 4.51 | / | 6.48 | 6.48 | / | 3.46 | 3.46 | / | 31º12′46″, 116º48′56″ |
| Shangcheng batholith (SCH) | | | | | | | | | | |
| Granitoid§ | | | | | | | | | | |
| 01SCH01 | 6.11, 6.05 | 6.08 | 0.04 | 9.08 | 9.08 | / | 7.83 | 7.83 | / | 31º47′54″, 115º21′28″ |
| 01SCH02 | 4.62 | 4.62 | / | 9.27, 9.28 | 9.28 | 0.01 | 7.60, 7.58 | 7.59 | 0.01 | 31º49′03″, 115º27′20″ |
| Sidaohe | | | | | | | | | | |
| Gneiss# | | | | | | | | | | |
| 00DB63 | -0.59, -0.67, -0.64 | -0.63 | 0.04 | 3.11, 2.91 | 3.01 | 0.14 | 1.77 | 1.77 | / | 31º22′12″, 115º04′09″ |
| 00DB64 | -1.75, -1.51 | -1.63 | 0.17 | 2.53, 2.51 | 2.52 | 0.01 | 1.22 | 1.22 | / | 31º22′12″, 115º04′09″ |

| | | | | | | | | | | |
|---|---|---|---|---|---|---|---|---|---|---|
| Tianzhushan/Yuexi pluton (TZS)** | | | | | | | | | | |
| Granitoid†† | | | | | | | | | | |
| 03TZ01 | 4.98 | 4.98 | / | 8.14 | 8.14 | / | 7.00 | 7.00 | / | 30º50′41″, 116º17′14″ |
| 03TZ02 | 5.18 | 5.18 | / | 8.01 | 8.01 | / | 7.08 | 7.08 | / | 30º50′31″, 116º18′09″ |
| 03TZ03 | 5.57 | 5.57 | / | 8.53 | 8.53 | / | 5.99 | 5.99 | / | 30º50′39″, 116º19′05″ |
| 03TZ05 | 5.83 | 5.83 | / | 9.16 | 9.16 | / | 7.80 | 7.80 | / | 30º48′25″, 116º20′45″ |
| 03TZ06 | 5.54 | 5.54 | / | / | / | / | 3.76 | 3.76 | / | 30º46′41″, 116º20′45″ |
| 03TZ08 | 5.56 | 5.56 | / | 8.50 | 8.50 | / | 2.14 | 2.14 | / | 30º45′42″, 116º20′34″ |
| 03TZ09 | 5.44 | 5.44 | / | 8.81 | 8.81 | / | 7.40 | 7.40 | / | 30º44′20″, 116º22′02″ |
| 03TZ10 | 5.14 | 5.14 | / | 7.95 | 7.95 | / | 6.50 | 6.50 | / | 30º43′25″, 116º23′08″ |
| 03TZ11 | 5.40 | 5.40 | / | 8.33 | 8.33 | / | 6.69 | 6.69 | / | 30º43′23″, 116º26′52″ |
| 03TZ12 | 5.08 | 5.08 | / | 7.93 | 7.93 | / | 6.27 | 6.27 | / | 30º45′22″, 116º26′06″ |
| 03TZ16 | 4.41 | 4.41 | / | 7.48 | 7.48 | / | 4.18 | 4.18 | / | 30º43′51″, 116º28′02″ |
| 03TZ17 | 5.09 | 5.09 | / | 7.98 | 7.98 | / | 6.44 | 6.44 | / | 30º44′26″, 116º27′11″ |
| 03TZ18 | 4.94 | 4.94 | / | 7.85 | 7.85 | / | 6.47 | 6.47 | / | 30º44′35″, 116º27′07″ |

| | | | | | | | | | | |
|---|---|---|---|---|---|---|---|---|---|---|
| 03TZ19 | 5.47 | 5.47 | / | 8.28 | 8.28 | / | 6.22 | 6.22 | / | 30º44′33″, 116º27′27″ |
| 03TZ20 | 5.29 | 5.29 | / | 8.17 | 8.17 | / | 6.10 | 6.10 | / | 30º43′56″, 116º27′26″ |
| 03TZ22 | 5.44 | 5.44 | / | 8.26 | 8.26 | / | 7.58 | 7.58 | / | 30º44′46″, 116º29′08″ |
| 02TZ01 | 5.32 | 5.32 | / | 7.90 | 7.90 | / | 5.82 | 5.82 | / | 30º43′45″, 116º26′49″ |
| 02TZ02 | 5.37 | 5.37 | / | 8.36 | 8.36 | / | 6.70 | 6.70 | / | 30º43′40″, 116º26′47″ |
| 02TZ03 | 5.17 | 5.17 | / | 8.18 | 8.18 | / | 6.02 | 6.02 | / | 30º43′28″, 116º26′53″ |
| 02TZ04 | 5.37 | 5.37 | / | 8.26 | 8.26 | / | 3.35 | 3.35 | / | 30º43′22″, 116º27′19″ |
| 02TZ05 | 5.00 | 5.00 | / | 7.96 | 7.96 | / | 2.32 | 2.32 | / | 30º43′38″, 116º27′47″ |
| Gneiss[#] | | | | | | | | | | |
| 01TZS05 | 0.33, 0.31 | 0.32 | 0.01 | 9.33 | 9.33 | / | 7.06 | 7.06 | / | 30º43′24″, 116º26′51″ |
| 01TZS07 | -3.78, -3.71 | -3.75 | 0.05 | 0.29, 0.14, 0.34 | 0.26 | 0.10 | -0.26, -0.01 | -0.14 | 0.18 | 30º42′06″, 116º29′13″ |

**Table S1.** Oxygen isotopes of granitoids and gneisses from the Dabie orogen in central-eastern China[*]. [*]Pluton and batholith are alphabetically tabulated throughout this study. [†]Data from Refs.[22,23]. [§]Data after Ref.[24]. [#]Data from Refs.[20,21,24]. [**]Abbreviation within parenthesis is labelled in Fig. 1, and that after / denotes alternative name adopted by other authors. [††]Data after Refs.[73,75].
